# Association between habitual intake of fermented milk products containing lactic acid bacteria and all-cause mortality: a community-based cohort prospective study

**DOI:** 10.1016/j.jnha.2026.100956

**Published:** 2026-08-18

**Authors:** Yukitoshi Aoyagi, Ryuta Amamoto, Sungjin Park, Kazuhito Shimamoto, Takashi Suwa, Hiroshi Makino, Satoshi Matsubara

**Affiliations:** a Exercise Sciences Research Group, Tokyo Metropolitan Institute of Gerontology, Itabashi, Tokyo, Japan; b Food Research Department, Yakult Central Institute, Kunitachi, Tokyo, Japan

**Keywords:** Probiotics, Fermented milk product, Mortality, Nakanojo study

## Abstract

**Background:**

Dairy product consumption is associated with a lower risk of all-cause mortality. However, the evidence regarding probiotic-fermented milk products remains unclear. This study examined the association between the intake of fermented milk products containing lactic acid bacteria, including *Lacticaseibacillus paracasei* strain Shirota (LcS), and all-cause mortality.

**Methods:**

A total of 1,280 community-dwelling Japanese aged ≥65 years (519 men, 761 women; mean age 73.5 years) were included in the analyses. Participants were categorized in two groups based on the retrospectively recalled intake frequency of fermented milk products (<3 or ≥3 days/week) over 5- and 10-year periods. All-cause mortality risk was evaluated using Cox proportional hazards regression analyses in the full sample and propensity score-matched subsamples.

**Results:**

During the 9.3-year follow-up period (112.05 months), deaths occurred in 147 men (28.3%) and 114 women (15.0%). Among men, consumption of fermented milk products containing LcS for ≥3 days/week over five years was significantly associated with a lower risk of all-cause mortality in both full sample analysis (hazard ratio [HR] 0.648, 95% confidence interval [CI] 0.438–0.958, p = 0.030) and matched analysis (HR 0.640, 95% CI 0.434–0.943, p = 0.024), compared with those consuming <3 days/week. Similar associations were observed for overall fermented milk products, including the LcS products. No associations were found among women for either LcS or overall fermented milk products.

**Conclusion:**

Habitual intake of fermented milk products containing LcS was associated with a lower risk of all-cause mortality in older Japanese men.

## Highlights

• LcS fermented milk intake was associated with a lower mortality risk in older men.
• Fermented milk product intake was not associated with mortality in women.
• The health benefits of LcS may collectively contribute to a reduced mortality risk.

## 1 Introduction

The global population is rapidly ageing, including in developing regions [1], making the extension of both life expectancy and healthy life expectancy among older adults a major societal challenge. Since 2000, we have conducted an epidemiological study among community-dwelling older adults aged ≥65 years in Nakanojo Town, Japan (the Nakanojo Study), assessing the relationships between daily physical activity and health [[2], [3], [4], [5]]. Since 2014, we have monitored the intake frequency of fermented milk products and examined the relationship between probiotic intake and health outcomes among older adults.

Probiotics are defined by the World Health Organization /Food and Agriculture Organization of the United Nations as live microorganisms which confer a health benefit when administered in adequate amounts. *Lacticaseibacillus paracasei* strain Shirota (LcS), previously known as *Lactobacillus casei* strain Shirota, is one of the most widely consumed probiotic strains worldwide. LcS reaches the intestine alive [[6], [7], [8], [9], [10]] and helps maintain microbiota balance [9,11,12]. LcS improves intestinal motility [9,12], immunoregulation [13,14], protection against infection [11,[15], [16], [17]], alleviates stress [[18], [19], [20]], and enhances sleep quality [21]. In our study, habitual consumption of fermented milk products containing LcS (LcS products) was also associated with a lower risk of hypertension [22], anaemia [23], and infrequent bowel movements [24]. As these conditions increase mortality risk, regular LcS consumption may also be associated with all-cause mortality.

Nutritional factors, including sodium [25], plant-based protein [26], vitamin D [27], and dietary fibre [28], as well as lifestyle behaviours, such as smoking [29] and alcohol consumption [30], have been associated with all-cause mortality. However, two meta-analyses found no clear link between dairy consumption and all-cause mortality [31,32]. Variations in dairy intake across populations may explain these inconsistent findings, indicating the need for country- or region-specific analyses. In Japan, where dairy intake is generally lower than in Western countries [33], higher consumption of milk, yoghurt, and other dairy products has been associated with reduced all-cause mortality [34,35]. However, dairy products represent heterogeneous exposures, including fermented dairy products, probiotic-containing products, and those containing specific probiotic strains. Previous studies often grouped probiotic-containing dairy products with those that did not contain probiotics, and the probiotic strains were not standardised. Because the physiological effects of probiotics are strain-specific, products containing specific strains, such as LcS, may have distinct effects. To clarify the potential benefits of probiotic intake on all-cause mortality, examining its association with dairy products containing a defined probiotic strain is necessary.

In this study, we investigated the relationship between all-cause mortality and the consumption of LcS products among older Japanese individuals.

## 2 Material and methods

### 2.1 Participants

The participants were self-supporting, independent Japanese volunteers aged 65 years or older, who had been recruited for the Nakanojo Study [[2], [3], [4], [5]]. Recruitment criteria included attendance at an annual medical examination, functional independence, and the absence of chronic or progressive conditions that could limit physical activity or have a major effect on an individual’s perceived quality of life (e.g. cancer, arthritic diseases, Parkinson’s disease, Alzheimer’s disease, multiple sclerosis, amyotrophic lateral sclerosis, and dementia). A total of 1,280 adults aged ≥65 years (519 men, 761 women; mean age, 73.5 years) were selected from participants of the Nakanojo Study (2014–2018) with complete data on the core variables: age, sex, body mass index (BMI), current smoking status, current alcohol consumption, and intake frequency of fermented milk products containing lactic acid bacteria. The process of participant selection is shown in Supplementary Fig. S1. This study was conducted in accordance with the ethical principles of the Declaration of Helsinki. After receiving full explanation, participants provided written informed consent for this ethics-approved study by the Tokyo Metropolitan Institute of Gerontology.

### 2.2 Follow up

Participants were followed up through the residential registry from the date of enrolment until the date of death, relocation from Nakanojo Town, or the end of the study (December 31, 2025), whichever occurred first. Vital status and migration were verified, and the dates of death and migration were recorded. Information on deaths and relocation was obtained from official registry data and ascertained independently by the study investigators, who were not aware of the participants’ exposure status. Survival time was defined as the duration from enrolment to death or censoring. Censoring was determined based on the earlier occurrence of either the date of relocation or the conclusion of the study.

### 2.3 Estimation of the frequency of intake of fermented milk products

The intake frequency of LcS products was assessed once at baseline by a certified nutritionist in an interview using pictures of a series of LcS products, including ‘Yakult’, ‘Joie’, ‘Soful’, and ‘Pretio’ (Yakult Honsha, Tokyo, Japan), each of which contains 0.9–40 billion live LcS per bottle. Participants were asked how many days/week products of this type were consumed over the prior 5- and 10-year periods, based on recall of their past intake habits. The validity of this assessment was examined in a subsample, as described in Supplementary Information 1. The intake frequency of general fermented milk products (hereafter referred to as ‘overall fermented milk products’), including yoghurt and LcS-containing products described above, was assessed using the same procedure. Given the variability in LcS content across products, the exposure was defined based on consumption frequency and should be interpreted as representing habitual intake of LcS-containing products rather than a dose of probiotic bacterial counts. The distribution of the intake frequency of LcS products and overall fermented milk products in our population is summarised in Supplementary Table S1.

Participants were classified as consuming a bottle of the product 2 days/week or less (designated as the ‘<3 days/week’ group) or 3 days/week or more (designated as the ‘≥3 days/week’ group). This threshold was defined in accordance with our previous studies [22,23], in which the same cutoff was adopted based on the categorization used in the Framingham Heart Study [36].

### 2.4 Anthropometric characteristics, blood profiles, and medical history

The physical characteristics of each participant (age, height, body mass, BMI, fat-free mass, and muscle mass) were determined using standard anthropometric techniques [37]. Blood pressure was measured after 5 min of seated rest using an automatic sphygmomanometer (BP-103iII; Colin Medical Technology Co. Ltd., Komaki, Aichi, Japan). At least one further measurement was taken after a further 5-min rest if the initial reading suggested that an individual had become hypertensive (or, rarely, hypotensive). The biochemical profile of each participant (red blood cells, haemoglobin, haematocrit, triglycerides, high-density lipoprotein cholesterol, low-density lipoprotein cholesterol, glycosylated haemoglobin A1c, fasting blood sugar, glutamic oxaloacetic transaminase, glutamic pyruvic transaminase, γ-glutamyl transpeptidase, albumin, creatinine, uric acid concentrations, and estimated glomerular filtration rate) was measured using standard methods (Health Sciences Research Institute Inc., Yokohama, Japan). Medical histories were obtained from the doctors’ records.

### 2.5 Physical activity patterns and physical health

The average number of steps taken per day was obtained by tracking steps 24 h/day over a 1-month period using a uniaxial acceleration sensor (Lifecorder, Suzuken, Nagoya, Japan), as described previously [5]. The average number of steps taken per day and daily cumulative duration of moderate-intensity exercise (activity demanding an energy expenditure greater than 3 metabolic equivalents [METs]) were calculated for each participant. The preferred and maximum walking speeds were determined over a 5-m distance using a stopwatch (SVAE101; Seiko, Tokyo, Japan), as described previously [38]. Each participant completed two trials at both comfortable and maximum walking speeds, and the average velocities were recorded. Peak handgrip force of the dominant hand was assessed using a Smedley dynamometer (ES-100; Evernew, Tokyo, Japan). Two trials were performed, and the higher reading was recorded. Quantitative ultrasound measurements of the osteosonic index of the calcaneus were performed using an Achilles ultrasonic bone densitometer (AOS-100; Aloka, Musashino, Japan) as described previously [39].

### 2.6 Nutrient intake

The nutritional status of the participants was evaluated by nutritionists over a 1-week period using validated Version 3.5 of the Food Frequency Questionnaire Based on Food Groups (Kenpakusha, Bunkyo, Tokyo, Japan) [40], a 20-item questionnaire regarding the consumption of items from 29 food groups and 10 methods of food preparation. Based on the responses to this questionnaire, the daily consumption of energy, nutrients, and food groups was estimated 1–2 months before the start of the study. The estimated nutrients included proteins, lipids, carbohydrates, dietary fibre, saturated fatty acids, monounsaturated fatty acids, polyunsaturated fatty acids, cholesterol, sodium, calcium, and iron.

### 2.7 Statistical analyses

Version 4.4.2, R [41] software (https://cran.r-project.org/) was used throughout the study.

Participants were divided into two arbitrary groups (men and women) based on the intake frequency of overall fermented milk or LcS products (<3 and ≥3 days/week). The primary analysis was defined as the association between intake of LcS products and all-cause mortality over the past 5 years, using Cox proportional hazards regression models adjusted for age, BMI, smoking status, and alcohol consumption, consistent with our previous studies [22,23]. Analyses of overall fermented milk products, over the past 10-year period, propensity score–matched subsamples, and restricted mean survival time (RMST) were considered secondary or sensitivity analyses.

Analyses of covariance were used to assess independent differences between these two groups with respect to anthropometric measurements, physical activity, physical fitness, and calcaneal, nutritional, and blood variables after controlling for age. Chi-square tests were used to assess differences in the percentages of current smokers and current alcohol consumers and the prevalence of disease between the two groups. Fisher’s exact test was used to compare the crude mortality proportions without accounting for follow-up time or baseline age imbalances. Cox proportional hazards regression analyses were used to examine the independent relationships between fermented milk product intake and the estimated hazard ratios (HRs) and 95% confidence intervals (CIs) of all-cause mortality, after controlling for age, BMI, smoking status, and alcohol consumption. Adjusted survival curves derived from the Cox model were plotted using the survival::survfit function. RMST was estimated to quantify the average survival time up to a prespecified time point. To estimate the covariate-adjusted difference in the RMST between groups, we applied a regression-based approach implemented in the survRM2::rmst2 function [42,43]. The truncation time (τ) was set to 138.11 months, corresponding to the maximum follow-up period. To evaluate the robustness of baseline imbalances, we constructed a matched cohort based on age, BMI, smoking status, and alcohol consumption using propensity score matching. Propensity scores were estimated using logistic regression analysis based on baseline covariates (age, BMI, smoking status, and alcohol consumption). Participants were matched using 1:1 nearest-neighbour (calliper = 0.2 × SD of logit [PS]) matching without replacement. Within the matched cohort, unadjusted Cox proportional hazards models with robust standard errors clustered by match sets and RMST analyses were performed. Statistical significance was defined at 0.05.

## 3 Results

During the 9.3-year follow-up period (112.05 months), deaths occurred in 147 men (28.3%) and 114 women (15.0%). In Cox proportional hazards models adjusted for age, BMI, smoking status, and alcohol consumption, a statistically significant interaction between sex and intake of LcS products was observed, whereas a similar trend was found for overall fermented milk products (p for interaction = 0.034, 0.066, respectively). Therefore, the results are presented separately for men and women.

### 3.1 Men

#### 3.1.1 Participant characteristics

Among the 519 Japanese men aged 65 years or over (mean [SD] age 74.3 [6.8] years), 401 and 118 men consumed LcS products <3 and ≥3 days/week over the past 5 years, respectively (Supplementary Table S2), and 246 and 273 men consumed overall fermented milk products <3 and ≥3 days/week, respectively (Supplementary Table S3). Compared with men consuming LcS products <3 days/week over the past 5 years, those consuming such products ≥3 days/week were significantly older at baseline and showed a lower prevalence of diabetes (Supplementary Table S2). Similar patterns were observed when the intake frequency was based on a 10‑year period (Supplementary Table S4).

Men consuming overall fermented milk products ≥3 days/week had fewer smokers, lower blood glucose, HbA1c and diabetes prevalence, and higher walking speed, calcaneal osteosonic index, muscle mass, and certain nutrient intakes than those in the <3 days/week group (Supplementary Table S3).

#### 3.1.2 Relationship between intake of fermented milk products and all‑cause mortality

**Fig. 1:**
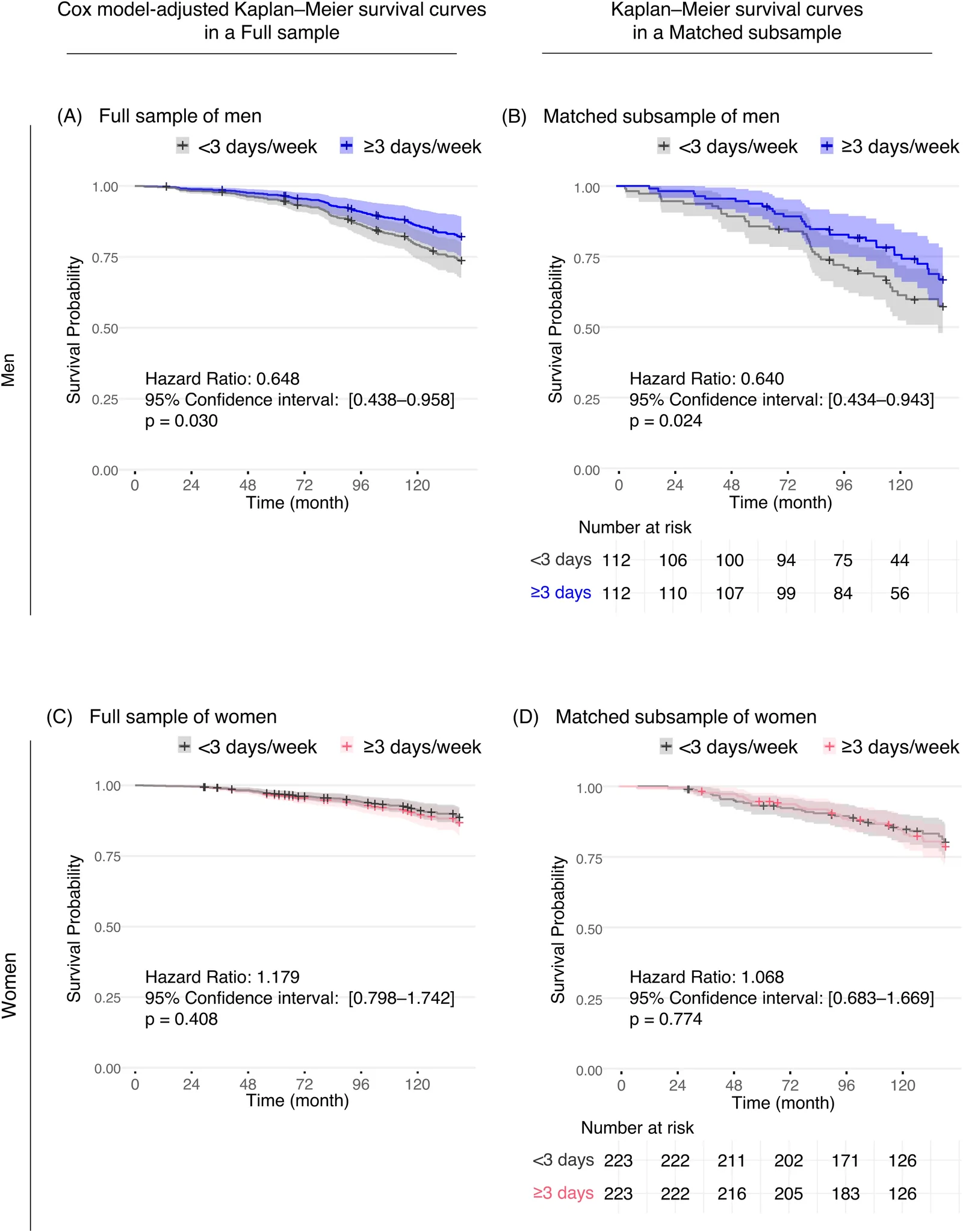
Kaplan–Meier survival curves for all-cause mortality in men (A, B) and women (C, D) consuming LcS products for <3 and ≥3 days/week over the past 5 years. (A, C) Cox model-adjusted Kaplan–Meier survival curves were obtained using Cox proportional hazards models, with age, BMI, smoking status, and alcohol consumption as covariates. (B, D) Kaplan–Meier survival curves in a matched subsample were obtained using propensity score–matching analyses balancing age, BMI, alcohol consumption, and smoking status. “+” symbols on the curves indicate censoring, and the shaded areas represent 95% confidence intervals. LcS, *Lacticaseibacillus paracasei* strain Shirota. Hazard ratios are shown for ≥3 days/week compared with <3 days/week. The numbers at risk cannot be directly defined for the adjusted survival curves derived from the multivariable Cox models, as these represent model-based predictions for a hypothetical population.

Crude analysis showed no significant differences in mortality rates between the <3 and ≥3 days/week groups for the LcS products. Unadjusted Kaplan–Meier curves are presented in Supplementary Fig. S2A. Although median follow-up time did not significantly differ between the intake groups (Supplementary Table S5, p = 0.280), baseline age differed between the intake groups (Supplementary Table S2, Fig. S3A). After adjustment for age, BMI, alcohol consumption, and smoking status, Cox proportional hazards regression models, with no violation of the proportional hazards assumption (global test p = 0.360), indicated that the HR for all‑cause mortality in the men consuming LcS products ≥3 days/week over the past 5 years was 0.648 (95% CI 0.438–0.958, p = 0.030), indicating a statistically significant association compared with that in men consuming <3 days/week (Table 1, Fig. 1A). Although this difference was not significant, a similar trend was observed in analyses of intake over the previous 10 years (Supplementary Table S6).

**Table 1:** Risk of all-cause mortality in men consuming LcS and overall fermented milk products for <3 or ≥3 days/week.

	Number of participants	Number of deaths	Mortality	Fisher’s test	Cox model		
			(%)	p value	Hazard Ratio	95% Confidence interval	p value
Full sample^a^							
LcS products over the past 5 years							
<3 days/week	401	113	28.2	–	1.000	–	–
≥3 days/week	118	34	28.8	0.908	0.648	[0.438–0.958]	**0.030**
Overall fermented milk products over the past 5 years							
<3 days/week	246	78	31.7	–	1.000	–	–
≥3 days/week	273	69	25.3	0.119	0.690	[0.499–0.955]	**0.025**

Matched subsample^b^							
LcS products over the past 5 years							
<3 days/week	112	42	37.5	–	1.000	–	–
≥3 days/week	112	30	26.8	0.115	0.640	[0.434–0.943]	**0.024**
Overall fermented milk products over the past 5 years							
<3 days/week	227	75	33.0	–	1.000	–	–
≥3 days/week	227	57	25.1	0.079	0.677	[0.499–0.920]	**0.013**

LcS, *Lacticaseibacillus paracasei* strain Shirota.

a Intergroup differences in the relative risk of all-cause mortality were assessed using Cox proportional hazards regression analysis adjusted for age, body mass index, smoking status, and alcohol consumption.

b Intergroup differences in the relative risk of all-cause mortality were assessed using unadjusted Cox proportional hazards models with robust standard errors clustered by matching sets.

Additional sensitivity analyses using intake frequency as a continuous variable showed a similar inverse association between the intake frequency of LcS products over the past 5 years and all-cause mortality (Supplementary Table S7; HR 0.931, 95% CI 0.872–0.995, p = 0.035). Furthermore, although no clear differences in baseline characteristics were observed between the two LcS intake groups except for age and diabetes prevalence (Supplementary Table S2), we conducted additional analyses adjusting for potential confounding factors. These included variables that showed differences between the intake groups of overall fermented milk products (e.g., muscle mass, preferred walking speed, osteosonic index, glycosylated haemoglobin A1c, and diabetes), as well as factors commonly associated with all-cause mortality (e.g., preferred walking speed, step count, estimated glomerular filtration rate, albumin, diabetes, and hypertension) (Supplementary Table S8). When nutrient intakes were additionally included in the model, the association became non-significant (Model 5, p = 0.160). However, a similar non-significant result was observed even when the analysis was restricted to participants with available nutrient intakes but without adjusting for these variables (Model 4, p = 0.141). This suggests that the attenuation of the association after dietary adjustment may reflect both the reduced sample size and residual dietary confounding. Despite the reduced sample size due to the inclusion of these variables, similar inverse associations were observed. Furthermore, sensitivity analyses excluding participants with follow-up shorter than 12–24 months yielded similar results, suggesting a limited impact of reverse causation (Supplementary Table S9).

For overall fermented milk products, including LcS products, the adjusted HR for all-cause mortality was 0.690 (95% CI 0.499–0.955, p = 0.025) for ≥3 versus <3 days/week (Table 1, Supplementary Fig. S4A). Although, a similar inverse association between the continuous intake frequency of overall fermented milk products and all-cause mortality was observed (Supplementary Table S7), the association was attenuated and did not remain statistically significant after additional adjustment for potential confounders in the restricted analytic sample (Supplementary Table S10).

#### 3.1.3 Relationship between intake of fermented milk products and RMST

The RMST, defined as the average survival time up to a prespecified time point, was estimated to evaluate differences in the average survival time between the two intake groups. Although not statistically significant, participants consuming LcS products ≥3 days/week had 4.51 months longer survival than that in the <3 days/week group (Table 2; 95% CI −1.99–11.02, p = 0.174). Similar results were observed for overall fermented milk products (Table 2). The results of the analysis based on the intake frequency over the past 10 years are presented in Supplementary Table S11.

**Table 2:** Restricted mean survival time in men consuming LcS and overall fermented milk products for <3 or ≥3 days/week.

	Number of participants	Mean difference in survival (months) (≥3 days/week − <3 days/week)	95% Confidence interval	p value
Full sample^a^				
LcS products	519	4.51	[−1.99 to 11.02]	0.174
Overall fermented milk products	519	4.45	[−1.18 to 10.08]	0.121

Matched subsample^b^				
LcS products	224	9.80	[0.656–18.95]	**0.036**
Overall fermented milk products	454	4.55	[−1.28 to 10.38]	0.126

LcS, *Lacticaseibacillus paracasei* strain Shirota.

a Intergroup differences in the restricted mean survival time up to 138.11 months, corresponding to the maximum follow-up period, were assessed after adjusting for age, body mass index, smoking status, and alcohol consumption.

b Intergroup differences in the restricted mean survival time up to 138.11 months were assessed using an unadjusted RMST model.

#### 3.1.4 Sensitivity analyses (Propensity score–matched subsample)

Standardised mean differences (SMDs) were used to assess covariate balance between ≥3 and <3 days/week groups. After 1:1 propensity score matching for age, BMI, alcohol consumption, and smoking status, all SMDs decreased to <0.1, indicating satisfactory balance (Supplementary Table S12). In addition, the distributions of key clinical and functional variables were largely similar between the full sample and the matched subsample (Supplementary Table S13), suggesting that the matched sample retained broadly comparable baseline characteristics to those of the original cohort. Among matched men, HR for all‑cause mortality in the group consuming LcS products ≥3 days/week over the past 5 years was 0.640 (Table 1, Fig. 1B; 95% CI 0.434–0.943, p = 0.024) with 9.8 months longer survival (Table 2; 95% CI 0.656–18.95, p = 0.036). For overall fermented milk products, HR patterns were similar, although the RMST differences were not significant (Table 1, Supplementary Fig. S4B, Table 2). Ten-year intake analyses (Supplementary Tables S6 and S11) showed similar trends.

### 3.2 Women

#### 3.2.1 Participant characteristics

Among 761 Japanese women aged ≥65 years (mean [SD] age 73.3 [6.4] years), 537 and 224 women consumed LcS products <3 and ≥3 days/week over the past 5 years, respectively (Supplementary Table S14), and 226 and 535 women consumed overall fermented milk products <3 and ≥3 days/week, respectively (Supplementary Table S15). Women with LcS intake <3 days/week over the past 5 years were significantly older and had lower physical activity, measured by daily steps and moderate-intensity activity duration (Supplementary Table S14). Similar trends were observed for intake frequency over the past 10 years (Supplementary Table S16).

For overall fermented milk products, women with intake ≥3 days/week had significantly higher preferred walking speed, greater handgrip strength, and higher nutrient intake levels than those in women with intake <3 days/week, whereas estimated glomerular filtration rate (eGFR) was significantly lower in the ≥3 days/week group (Supplementary Table S15).

#### 3.2.2 Relationship between intake of fermented milk products and all-cause mortality

Among women, crude mortality rates were marginally higher in the ≥3 days/week group, but these differences were not statistically significant. Unadjusted Kaplan–Meier curves are presented in Supplementary Fig. S2C. Similar to men, median follow-up time did not differ between the intake groups (Supplementary Table S17; p = 0.353), whereas baseline age differed between the intake groups (Supplementary Table S14, Fig. S3C). Adjusted Cox proportional hazards models showed a similar trend for all-cause mortality, though the association was non-significant after controlling for age, BMI, alcohol consumption and smoking (Table 3, Fig. 1C; HR 1.179, 95% CI 0.798–1.742, p = 0.408). Similarly, no association was observed between intake frequency as a continuous variable and the risk of all-cause mortality (Supplementary Table S18; HR 1.039, 95% CI 0.976–1.106, p = 0.230).

**Table 3:** Risk of all-cause mortality in women consuming LcS and overall fermented milk products for <3 or ≥3 days/week.

	Number of participants	Number of deaths	Mortality	Fisher’s test	Cox model		
			(%)	p value	Hazard Ratio	95% Confidence interval	p value
Full sample^a^							
LcS products over the past 5 years							
<3 days/week	537	74	13.8	–	1.000	–	–
≥3 days/week	224	40	17.9	0.181	1.179	[0.798–1.742]	0.408
Overall fermented milk products over the past 5 years							
<3 days/week	226	34	15.0	–	1.000	–	–
≥3 days/week	535	80	15.0	1.000	1.152	[0.766–1.732]	0.496

Matched subsample^b^							
LcS products over the past 5 years							
<3 days/week	223	36	16.1	–	1.000	–	–
≥3 days/week	223	39	17.5	0.800	1.068	[0.683–1.669]	0.774
Overall fermented milk products over the past 5 years							
<3 days/week	220	31	14.1	–	1.000	–	–
≥3 days/week	220	35	15.9	0.689	0.970	[0.624–1.507]	0.891

LcS, *Lacticaseibacillus paracasei* strain Shirota.

a Intergroup differences in the relative risk of all-cause mortality were assessed using Cox proportional hazards regression analysis adjusted for age, body mass index, smoking status, and alcohol consumption.

b Intergroup differences in the relative risk of all-cause mortality were assessed using unadjusted Cox proportional hazards models with robust standard errors clustered by matching sets.

Analyses based on the intake frequency of overall fermented milk products also showed no significant differences in the HRs between the two groups (Table 3 and Supplementary Fig. S4C).

#### 3.2.3 Relationship between intake of fermented milk products and RMST

In the RMST analyses adjusted for age, BMI, alcohol consumption, and smoking status in women, the average survival time up to τ = 138.11 months did not differ significantly between the <3 and ≥3 days/week for either LcS or overall fermented milk products (Table 4).

**Table 4:** Restricted mean survival time in women consuming LcS and overall fermented milk products for <3 or ≥3 days/week.

	Number of participants	Mean difference in survival (months) (≥3 days/week − <3 days/week)	95% Confidence interval	p value
Full sample^a^				
LcS products	761	−1.71	[−5.65 to 2.24]	0.396
Overall fermented milk products	761	−1.37	[−5.08 to 2.33]	0.467

Matched subsample^b^				
LcS products	446	0.24	[−4.66 to 5.14]	0.923
Overall fermented milk products	440	−1.24	[−5.69 to 3.22]	0.586

LcS, *Lacticaseibacillus paracasei* strain Shirota.

a Intergroup differences in the restricted mean survival time up to 138.11 months, corresponding to the maximum follow-up period, were assessed after adjusting for age, body mass index, smoking status, and alcohol consumption.

b Intergroup differences in the restricted mean survival time up to 138.11 months were assessed using an unadjusted RMST model.

#### 3.2.4 Sensitivity analyses (Propensity score–matched subsample)

In propensity score–matched analyses balancing age, BMI, alcohol consumption, and smoking status, although all SMDs were decreased to below 0.1 (Supplementary Table S19), no significant differences were observed in women between the <3 and ≥3 days/week groups with respect to the risk of all-cause mortality or survival time for LcS products (Table 3, Fig. 1D, Table 4).

Similarly, the intake frequency of overall fermented milk products showed no statistically significant association with mortality or survival time in the matched subsamples (Table 3, Supplementary Fig. S4D, Table 4).

## 4 Discussion

In the primary analysis adjusted for age, BMI, alcohol consumption, and smoking status, men who consumed LcS-containing fermented milk products ≥3 days/week over the past 5 years had a significantly lower risk of all-cause mortality than that in those consuming <3 days/week (Table 1, Fig. 1A; HR 0.648, p = 0.030).

The difference between the crude mortality rates and adjusted hazard ratios may be explained by differences in the baseline age between the intake groups. Although there was no difference in the crude mortality rate and follow-up period between the groups, the baseline age differed. In a model including age as a confounding variable, the association between LcS intake and all-cause mortality became apparent. These results suggest that the observed association is explained by the difference in baseline age between the intake groups and is unlikely to be an artifact of the model specification.

To address potential residual confounding due to ‘healthy user bias’, whereby individuals who consume probiotic products may have higher health awareness, additional sensitivity analyses were conducted with adjustments for potential confounding factors, such as physical health, physical activity, blood profile, and dietary intake. Although the number of participants was reduced, LcS product intake remained inversely associated with all-cause mortality. In addition, to address potential ‘reverse causation’, whereby healthier individuals may be more likely to consume LcS products, additional sensitivity analyses were performed excluding deaths occurring within the first 12–24 months, which yielded similar results. These findings reduce concerns that bias may have influenced the observed association. However, residual confounding related to dietary factors and other unmeasured factors cannot be excluded.

Furthermore, the propensity score-matched sensitivity analysis balancing age and other baseline characteristics also showed a significantly lower risk in the ≥3 days/week group (Table 1, Fig. 1B; HR 0.640, p = 0.024). Analyses in which participants were grouped according to their intake frequency over the past 10 years showed similar tendencies, although the results were not statistically significant.

Taken together, these findings suggest that consuming LcS products on ≥3 days/week over the past 5 years was associated with lower risk of all-cause mortality among older men, indicating a possible link between LcS intake and mortality risk.

In contrast, for overall fermented milk products, although a statistically significant inverse association was observed in analysis adjusted for major confounders (age, BMI, alcohol consumption, and smoking), this association was attenuated and did not remain statistically significant after additional adjustment for potential confounders in the restricted analytic sample, suggesting the possibility of residual confounding.

Regarding survival time, men consuming LcS products ≥3 days/week showed a longer RMST than that in those consuming <3 days/week, with an increase of 4.51 months in the full sample (Table 2; p = 0.174) and 9.80 months in the propensity score-matched subsample (Table 2; p = 0.036). Because the RMST captures differences in survival by integrating the accumulated separation of survival curves over a prespecified time horizon, the discrepancy from the Cox proportional hazards model results is likely attributable to the relatively short follow-up period, which limits the degree of divergence between the survival curves. Extending the follow-up period in future studies is expected to elucidate the differences in RMST across all participants included in the analysis. Although the current data do not conclusively demonstrate a definitive extension of survival for all LcS consumers, they suggest a potential association with longer survival, which may be related to habitual consumption.

Among women, the multivariable-adjusted models and propensity score-matched analyses did not demonstrate significant associations between fermented milk intake and the risk of all-cause mortality (Table 3, Fig. 1C and D). Previous large-scale Japanese studies have similarly reported that higher consumption of dairy products, including fermented milk, correlates with a lower risk of all-cause mortality in men, whereas no such association was observed in women [35], which is consistent with our observations.

One potential explanation for these sex-specific patterns is that women generally have healthier lifestyle profiles than those in men, which may attenuate the association between dairy intake and mortality [35]. In our cohort, women had lower prevalences of current smoking, alcohol consumption, hypertension, and diabetes than men (Supplementary Table S20), potentially making the detection of any incremental benefits of fermented milk intake more challenging. Furthermore, the overall mortality rate was lower in women (15.0%) than in men (28.3%), and the gap between the expected age at death (based on life tables) and observed age at death tended to be larger in women (Supplementary Table S21). Complementary analyses may provide further context for the findings in women. When women with baseline characteristics comparable to those of men (e.g., age, BMI, smoking status, alcohol consumption, and prevalence of major disease) were selected using propensity score matching, overall mortality increased and the estimated hazard ratio tended to decrease (Supplementary Table S22). When the analyses were further restricted to women with longer follow-up periods, a gradual decrease in the estimated hazard ratios was observed with increasing follow-up duration (Supplementary Table S23). These observations suggest that differences in baseline risk and follow-up duration may partly explain the observed differences between men and women, and that a longer follow-up period may be necessary to evaluate the mortality associations in women with sufficient power.

Several mechanisms may underlie this association observed in men. The habitual consumption of LcS products is associated with a lower risk of incident hypertension [22] and anaemia [23], both of which may contribute to cardiovascular and ischaemic outcomes. LcS reaches the intestine alive [[6], [7], [8], [9], [10]] and exerts beneficial effects on intestinal function, such as lowering the pH in the intestine and providing a good balance of gut microbiota by increasing the abundance of beneficial bacteria and decreasing the abundance of harmful bacteria [9,11,12]. Constipation has been associated with an increased risk of cardiovascular disease and all-cause mortality [[44], [45], [46]]. In addition, dysbiosis related to constipation or diarrhoea and the accompanying changes in gut-derived metabolites may impair renal function [47], intestinal barrier function and promote chronic inflammation [[48], [49], [50]]. These mechanisms may be involved in the observed association, although they were not directly examined in the present cohort. Moreover, LcS reduces the incidence or duration of common infections [11,[15], [16], [17]], which in turn might lower the risk of severe outcomes such as pneumonia. Previous findings indicated that LcS might have preventive effects against bladder and colorectal cancers [51,52]. Collectively, these diverse health effects may be related to the observed association; however, additional evidence, particularly from other prospective cohorts and randomised controlled trials, will be required to establish the usefulness of LcS‑containing products for longevity.

The study has certain limitations. Individuals consuming LcS products ≥3 days/week may engage in healthy behaviours. Despite examining many lifestyle covariates, differences were minimal, though diabetes incidence was lower in the <3 days/week group. The inverse association remained even after additional adjustment for diabetes history and propensity score–matched analysis, but residual confounding factors such as diet quality, dietary patterns, educational attainment, income, family structure, and changes in intake habits during follow-up, could not be entirely dismissed. Therefore, the observed association may partly reflect broader lifestyle patterns and socioeconomic circumstances rather than a direct effect of the exposure.

The assessment of intake of fermented milk products was based on retrospective self-report and may be subject to recall bias. A validation analysis in a subsample showed high agreement between retrospectively reported and prospectively derived intake frequencies over 5 years, although this was conducted in a relatively healthy subgroup.

In addition, the variability in LcS bacterial counts across products could not be accounted for due to the lack of detailed product-specific intake frequency. Therefore, the exposure in this study should be interpreted as a measure of habitual intake patterns rather than a precise quantitative estimate of the bacterial dose.

The absence of cause-of-death information restricts mechanistic inferences. Replication in other prospective cohorts and confirmation in randomised controlled trials are essential to establish the generalisability and causality.

Finally, the study population consisted of relatively healthy, community-dwelling older adults from a single region, with the exclusion of individuals with major chronic conditions. This may have introduced a healthy cohort effect and limited the variability in both exposures and outcomes. Further studies in independent cohorts are needed to assess the generalizability of these findings.

## 5 Conclusion

In this community-based cohort of older Japanese, habitual consumption of fermented milk products containing LcS was associated with a lower risk of all-cause mortality in older Japanese men.

## CRediT authorship contribution statement

Yukitoshi Aoyagi: Conceptualization, Methodology, Formal analysis, Data curation, Writing‑Original Draft, Writing‑Review & Editing, Supervision, Project administration, Funding acquisition. Ryuta Amamoto: Conceptualization, Methodology, Writing‑Original Draft, Writing‑Review & Editing. Sungjin Park: Investigation, Formal analysis, Writing‑Review & Editing. Kazuhito Shimamoto: Methodology, Writing‑Review & Editing. Takashi Suwa: Methodology, Writing‑Review & Editing. Hiroshi Makino: Methodology, Writing‑Review & Editing. Satoshi Matsubara: Conceptualization, Methodology, Writing‑Review & Editing, Funding acquisition.

## Consent for publication

Not applicable.

## Ethics approval and consent to participate

This study was conducted in accordance with the ethical principles of the Declaration of Helsinki and was approved by the ethics committee of the Tokyo Metropolitan Institute of Gerontology. After receiving a full explanation of the study objectives and procedures, all participants provided written informed consent prior to participation.

## Declaration of Generative AI and AI-assisted technologies in the writing process

AI was not used in the preparation of this manuscript nor its table or figures.

## Funding

This study was supported by grants [Grant-in-Aid for Encouragement of Young Scientists: 12770037 and Grant-in-Aid for Scientific Research (C): 15500503, (C): 17500493, (B): 19300235, and (B): 23300259] from the Japan Society for the Promotion of Science and grants from Yakult Honsha Co. Ltd. and the Tokyo Metropolitan Institute of Gerontology. Yukitoshi Aoyagi and Sungjin Park are affiliated with the Tokyo Metropolitan Institute of Gerontology; Ryuta Amamoto, Kazuhito Shimamoto, Takashi Suwa, Hiroshi Makino, and Satoshi Matsubara are affiliated with Yakult Honsha Co., Ltd.

## Data availability

The data and analytic code presented in this study will be made available on reasonable request from the corresponding author. The data are not publicly available due to restrictions of informed consent, ongoing related research and confidentiality considerations.

## Declaration of competing interest

The authors declare the following competing interests: This study was partially supported by Yakult Honsha Co., Ltd. Several authors (Ryuta Amamoto, Kazuhito Shimamoto, Takashi Suwa, Hiroshi Makino, and Satoshi Matsubara) are employees of Yakult Honsha Co., Ltd. The funder had no role in data collection, data interpretation, or the decision to submit the manuscript for publication.

## Footnotes

Appendix A Supplementary material related to this article can be found, in the online version, at doi:https://doi.org/10.1016/j.jnha.2026.100956.
