# Supplementary material for: Association between habitual intake of fermented milk products containing lactic acid bacteria and all-cause mortality: a community-based cohort prospective study
Source: J Nutr Health Aging. 2026 Aug 18;30(10):100956. doi: 10.1016/j.jnha.2026.100956 (PMC13508645; doi:10.1016/j.jnha.2026.100956)
Supplement: Supplementary file 1 [file mmc1.docx]

**Supplementary Tables and Figures**

**Association between habitual intake of fermented milk products containing lactic acid bacteria and all-cause mortality: a community-based cohort prospective study**

Yukitoshi Aoyagi^a*^

Ryuta Amamoto^b^

Sungjin Park^a^

Kazuhito Shimamoto^b^

Takashi Suwa^b^

Hiroshi Makino^b^

Satoshi Matsubara^b^

^a^ Exercise Sciences Research Group, Tokyo Metropolitan Institute of Gerontology, Itabashi, Tokyo, Japan

^b^ Food Research Department, Yakult Central Institute, Kunitachi, Tokyo, Japan

*Address correspondence to

Yukitoshi Aoyagi, Ph.D.

Exercise Sciences Research Group, Tokyo Metropolitan Institute of Gerontology

35-2 Sakaecho, Itabashi-ku, Tokyo 173-0015, Japan

E-mail: aoyagi@tmig.or.jp; Tel: +81-3-3964-3241 ext. 4263; Fax: +81-3-3579-4776

Supplementary Table S1. Frequency of consumption of fermented milk products containing LcS and overall fermented milk products among 1,280 older Japanese individuals

|  | Intake frequency over the past 5 years (days/week) | | | | | | | |
| --- | --- | --- | --- | --- | --- | --- | --- | --- |
|  | 0 | 1 | 2 | 3 | 4 | 5 | 6 | 7 |
| Men | | | | | | | | |
| LcS products | 321 | 53 | 27 | 28 | 9 | 6 | 0 | 75 |
| Overall fermented milk products | 147 | 57 | 42 | 51 | 22 | 9 | 1 | 190 |
| Women | | | | | | | | |
| LcS products | 416 | 78 | 43 | 40 | 17 | 12 | 3 | 152 |
| Overall fermented milk products | 88 | 74 | 64 | 83 | 38 | 27 | 8 | 379 |

LcS, *Lacticaseibacillus* *paracasei* strain Shirota.

Supplementary Table S2. Characteristics of men consuming LcS products <3 or ≥3 days/week over the past 5 years

|  | <3 days/week | | | |  | ≥3 days/week | | | |  | |
| --- | --- | --- | --- | --- | --- | --- | --- | --- | --- | --- | --- |
|  | n | Mean ± SD | | |  | n | Mean ± SD | | | p value |  |
| Anthropometric parameters |  |  |  |  |  |  |  |  |  |  |  |
| Age | 401 | 73.6 | ± | 6.4 |  | 118 | 76.7 | ± | 7.4 | **<0.001** |  |
| Height (m) | 401 | 1.63 | ± | 0.07 |  | 118 | 1.62 | ± | 0.06 | 0.751 |  |
| Body mass (kg) | 401 | 61.9 | ± | 8.7 |  | 118 | 60.1 | ± | 9.4 | 0.593 |  |
| Body mass index (kg/m^2^) | 401 | 23.3 | ± | 2.5 |  | 118 | 22.9 | ± | 3.2 | 0.428 |  |
| Fat-free mass (kg) | 390 | 48.9 | ± | 5.5 |  | 110 | 47.6 | ± | 5.3 | 0.481 |  |
| Muscle mass (kg) | 390 | 46.3 | ± | 5.3 |  | 110 | 45.1 | ± | 5.0 | 0.482 |  |
| Current smoking and drinking habits |  |  |  |  |  |  |  |  |  |  |  |
| Smoker (number [%]) | 401 | 53 |  | [13.2] |  | 118 | 13 |  | [11.0] | 0.528 |  |
| Alcohol consumer (number [%]) | 401 | 263 |  | [65.6] |  | 118 | 71 |  | [60.2] | 0.280 |  |
| Physical health measurements |  |  |  |  |  |  |  |  |  |  |  |
| Systolic blood pressure (mmHg) | 362 | 130.3 | ± | 16.6 |  | 96 | 131.8 | ± | 18.0 | 0.525 |  |
| Diastolic blood pressure (mmHg) | 362 | 78.9 | ± | 10.3 |  | 96 | 77.8 | ± | 12.0 | 0.933 |  |
| Preferred walking speed (m/s) | 382 | 1.36 | ± | 0.2 |  | 111 | 1.31 | ± | 0.2 | 0.456 |  |
| Maximal walking speed (m/s) | 380 | 2.11 | ± | 0.4 |  | 109 | 2.04 | ± | 0.4 | 0.586 |  |
| Peak handgrip force (N) | 388 | 347 | ± | 72 |  | 111 | 331 | ± | 77 | 0.954 |  |
| Calcaneal osteosonic index (×10^6^) | 395 | 2.55 | ± | 0.4 |  | 112 | 2.49 | ± | 0.4 | 0.678 |  |
| Habitual physical activity patterns |  |  |  |  |  |  |  |  |  |  |  |
| Step count (steps/day) | 215 | 7314 | ± | 3904 |  | 69 | 6539 | ± | 2830 | 0.578 |  |
| Duration of exercise > 3 METs (min/day) | 215 | 18.4 | ± | 17.2 |  | 69 | 14.1 | ± | 13.7 | 0.249 |  |
| Blood profile |  |  |  |  |  |  |  |  |  |  |  |
| Triglyceride (mmol/L) | 341 | 1.56 | ± | 0.92 |  | 91 | 1.52 | ± | 0.88 | 0.943 |  |
| High-density lipoprotein cholesterol (mmol/L) | 341 | 1.47 | ± | 0.44 |  | 91 | 1.47 | ± | 0.37 | 0.792 |  |
| Low-density lipoprotein cholesterol (mmol/L) | 341 | 2.89 | ± | 0.77 |  | 91 | 2.92 | ± | 0.72 | 0.433 |  |
| Glutamic oxaloacetic transaminase (IU/L) | 341 | 25.4 | ± | 18.7 |  | 91 | 24.3 | ± | 7.8 | 0.719 |  |
| Glutamic pyruvic transaminase (IU/L) | 341 | 21.9 | ± | 19.0 |  | 91 | 20.0 | ± | 8.0 | 0.586 |  |
| γ-Glutamyl transpeptidase (IU/L) | 341 | 39.1 | ± | 95.4 |  | 91 | 34.5 | ± | 28.4 | 0.896 |  |
| Glycosylated haemoglobin A1c (%) | 341 | 5.77 | ± | 0.6 |  | 91 | 5.66 | ± | 0.5 | 0.060 |  |
| Blood sugar (mmol/L) | 341 | 6.67 | ± | 2.11 |  | 91 | 6.41 | ± | 1.76 | 0.085 |  |
| Albumin (g/L) | 341 | 43.4 | ± | 2.8 |  | 91 | 43.1 | ± | 2.5 | 0.589 |  |
| Creatinine (μmol/L) | 341 | 82.8 | ± | 25.0 |  | 91 | 83.0 | ± | 37.7 | 0.532 |  |
| Uric acid (μmol/L) | 341 | 331 | ± | 74 |  | 91 | 333 | ± | 79 | 0.666 |  |
| Estimated glomerular filtration rate (mL/min/1.73m^2^) | 341 | 64.7 | ± | 13.8 |  | 91 | 65.6 | ± | 15.1 | 0.120 |  |
| Prevalence |  |  |  |  |  |  |  |  |  |  |  |
| Hypertension (number [%]) | 401 | 197 |  | [49.1] |  | 118 | 66 |  | [55.9] | 0.194 |  |
| Dyslipidaemia (number [%]) | 401 | 72 |  | [18.0] |  | 118 | 18 |  | [15.3] | 0.496 |  |
| Diabetes (number [%]) | 401 | 54 |  | [13.5] |  | 118 | 8 |  | [6.8] | **0.049** |  |
| Malignant neoplasms (number [%]) | 401 | 40 |  | [10.0] |  | 118 | 16 |  | [13.6] | 0.270 |  |
| Nutrient intakes |  |  |  |  |  |  |  |  |  |  |  |
| Energy (kcal/day) | 304 | 2095 | ± | 599 |  | 85 | 2253 | ± | 578 | 0.067 |  |
| Protein (g/day) | 304 | 73.8 | ± | 24.2 |  | 85 | 80.9 | ± | 28.5 | 0.076 |  |
| Lipid (g/day) | 304 | 65.9 | ± | 25.1 |  | 85 | 72.7 | ± | 30.7 | 0.086 |  |
| Carbohydrate (g/day) | 304 | 278 | ± | 79 |  | 85 | 292 | ± | 75 | 0.279 |  |
| Dietary fiber (g/day) | 304 | 16.2 | ± | 5.6 |  | 85 | 17.0 | ± | 5.0 | 0.466 |  |
| Saturated fatty acid (g/day) | 304 | 20.3 | ± | 8.2 |  | 85 | 22.1 | ± | 10.2 | 0.179 |  |
| Monounsaturated fatty acid (g/day) | 304 | 22.0 | ± | 8.8 |  | 85 | 24.1 | ± | 11.8 | 0.116 |  |
| Polyunsaturated fatty acid (g/day) | 304 | 14.0 | ± | 5.3 |  | 85 | 15.1 | ± | 5.8 | 0.159 |  |
| Cholesterol (mg/day) | 304 | 329 | ± | 137 |  | 85 | 356 | ± | 155 | 0.405 |  |
| Sodium (mg/day) | 304 | 4539 | ± | 1795 |  | 85 | 4880 | ± | 1737 | 0.329 |  |
| Potassium (mg/day) | 304 | 2788 | ± | 1013 |  | 85 | 2903 | ± | 922 | 0.624 |  |
| Calcium (mg/day) | 304 | 718 | ± | 272 |  | 85 | 780 | ± | 285 | 0.213 |  |
| Magnesium (mg/day) | 304 | 294 | ± | 96 |  | 85 | 311 | ± | 94 | 0.269 |  |
| Iron (mg/day) | 304 | 8.86 | ± | 3.26 |  | 85 | 9.79 | ± | 3.57 | 0.095 |  |
| Vitamin C (mg/day) | 304 | 112 | ± | 55 |  | 85 | 118 | ± | 50 | 0.667 |  |

LcS, *Lacticaseibacillus* *paracasei* strain Shirota; METs, metabolic equivalents; SD, standard deviation. Values are presented as mean ± SD or n (%). Intergroup differences in the ratios of smokers/non-smokers, alcohol consumers/non-consumers, and prevalence of disease were assessed by χ^2^ tests. Independent differences in the other variables between the groups were assessed using analysis of covariance after adjusting for age. Differences in age between groups were assessed using t-tests.

Supplementary Table S3. Characteristics of men consuming overall fermented milk products <3 or ≥3 days/week over the past 5 years

|  | <3 days/week | | | |  | ≥3 days/week | | | |  | |
| --- | --- | --- | --- | --- | --- | --- | --- | --- | --- | --- | --- |
|  | n | Mean ± SD | | |  | n | Mean ± SD | | | p value |  |
| Anthropometric parameters |  |  |  |  |  |  |  |  |  |  |  |
| Age | 246 | 73.9 | ± | 6.7 |  | 273 | 74.7 | ± | 6.9 | 0.175 |  |
| Height (m) | 246 | 1.62 | ± | 0.06 |  | 273 | 1.63 | ± | 0.07 | **0.002** |  |
| Body mass (kg) | 246 | 61.1 | ± | 8.5 |  | 273 | 61.8 | ± | 9.2 | 0.140 |  |
| Body mass index (kg/m^2^) | 246 | 23.3 | ± | 2.6 |  | 273 | 23.2 | ± | 2.8 | 0.798 |  |
| Fat-free mass (kg) | 240 | 48.2 | ± | 5.5 |  | 260 | 49.0 | ± | 5.5 | **0.037** |  |
| Muscle mass (kg) | 240 | 45.7 | ± | 5.2 |  | 260 | 46.4 | ± | 5.2 | **0.037** |  |
| Current smoking and drinking habits |  |  |  |  |  |  |  |  |  |  |  |
| Smoker (number [%]) | 246 | 41 |  | [16.7] |  | 273 | 25 |  | [9.2] | **0.010** |  |
| Alcohol consumer (number [%]) | 246 | 161 |  | [65.4] |  | 273 | 173 |  | [63.4] | 0.622 |  |
| Physical health measurements |  |  |  |  |  |  |  |  |  |  |  |
| Systolic blood pressure (mmHg) | 219 | 131.5 | ± | 16.8 |  | 239 | 129.8 | ± | 17.1 | 0.242 |  |
| Diastolic blood pressure (mmHg) | 219 | 79.1 | ± | 10.8 |  | 239 | 78.2 | ± | 10.6 | 0.587 |  |
| Preferred walking speed (m/s) | 235 | 1.32 | ± | 0.2 |  | 258 | 1.36 | ± | 0.2 | **0.010** |  |
| Maximal walking speed (m/s) | 233 | 2.06 | ± | 0.4 |  | 256 | 2.13 | ± | 0.4 | **0.022** |  |
| Peak handgrip force (N) | 239 | 343 | ± | 73 |  | 260 | 344 | ± | 74 | 0.359 |  |
| Calcaneal osteosonic index (×10^6^) | 242 | 2.50 | ± | 0.4 |  | 265 | 2.57 | ± | 0.4 | **0.016** |  |
| Habitual physical activity patterns |  |  |  |  |  |  |  |  |  |  |  |
| Step count (steps/day) | 128 | 7227 | ± | 3859 |  | 156 | 7042 | ± | 3541 | 0.716 |  |
| Duration of exercise > 3 METs (min/day) | 128 | 17.5 | ± | 16.7 |  | 156 | 17.1 | ± | 16.4 | 0.878 |  |
| Blood profile |  |  |  |  |  |  |  |  |  |  |  |
| Triglyceride (mmol/L) | 211 | 1.54 | ± | 0.96 |  | 221 | 1.56 | ± | 0.86 | 0.678 |  |
| High-density lipoprotein cholesterol (mmol/L) | 211 | 1.47 | ± | 0.47 |  | 221 | 1.47 | ± | 0.38 | 0.995 |  |
| Low-density lipoprotein cholesterol (mmol/L) | 211 | 2.86 | ± | 0.75 |  | 221 | 2.92 | ± | 0.77 | 0.327 |  |
| Glutamic oxaloacetic transaminase (IU/L) | 211 | 26.2 | ± | 23.3 |  | 221 | 24.1 | ± | 6.7 | 0.241 |  |
| Glutamic pyruvic transaminase (IU/L) | 211 | 22.2 | ± | 23.2 |  | 221 | 20.8 | ± | 8.4 | 0.511 |  |
| γ-Glutamyl transpeptidase (IU/L) | 211 | 44.2 | ± | 119.8 |  | 221 | 32.3 | ± | 25.0 | 0.186 |  |
| Glycosylated haemoglobin A1c (%) | 211 | 5.84 | ± | 0.7 |  | 221 | 5.66 | ± | 0.5 | **0.001** |  |
| Blood sugar (mmol/L) | 211 | 6.95 | ± | 2.42 |  | 221 | 6.29 | ± | 1.53 | **<0.001** |  |
| Albumin (g/L) | 211 | 43.2 | ± | 2.6 |  | 221 | 43.5 | ± | 2.8 | 0.131 |  |
| Creatinine (μmol/L) | 211 | 83.9 | ± | 29.6 |  | 221 | 81.8 | ± | 26.6 | 0.293 |  |
| Uric acid (μmol/L) | 211 | 328 | ± | 78 |  | 221 | 334 | ± | 71 | 0.360 |  |
| Estimated glomerular filtration rate (mL/min/1.73m^2^) | 211 | 64.7 | ± | 15.0 |  | 221 | 65.1 | ± | 13.2 | 0.483 |  |
| Prevalence |  |  |  |  |  |  |  |  |  |  |  |
| Hypertension (number [%]) | 246 | 127 |  | [51.6] |  | 273 | 136 |  | [49.8] | 0.681 |  |
| Dyslipidaemia (number [%]) | 246 | 36 |  | [14.6] |  | 273 | 54 |  | [19.8] | 0.122 |  |
| Diabetes (number [%]) | 246 | 37 |  | [15.0] |  | 273 | 25 |  | [9.2] | **0.039** |  |
| Malignant neoplasms (number [%]) | 246 | 24 |  | [9.8] |  | 273 | 32 |  | [11.7] | 0.471 |  |
| Nutrient intakes |  |  |  |  |  |  |  |  |  |  |  |
| Energy (kcal/day) | 177 | 2054 | ± | 567 |  | 212 | 2193 | ± | 617 | **0.030** |  |
| Protein (g/day) | 177 | 70.5 | ± | 23.0 |  | 212 | 79.4 | ± | 26.4 | **<0.001** |  |
| Lipid (g/day) | 177 | 62.6 | ± | 23.7 |  | 212 | 71.3 | ± | 28.2 | **0.002** |  |
| Carbohydrate (g/day) | 177 | 277 | ± | 76 |  | 212 | 284 | ± | 81 | 0.403 |  |
| Dietary fiber (g/day) | 177 | 15.4 | ± | 5.0 |  | 212 | 17.1 | ± | 5.8 | **0.004** |  |
| Saturated fatty acid (g/day) | 177 | 19.0 | ± | 7.9 |  | 212 | 22.0 | ± | 9.2 | **0.001** |  |
| Monounsaturated fatty acid (g/day) | 177 | 21.0 | ± | 8.3 |  | 212 | 23.7 | ± | 10.3 | **0.008** |  |
| Polyunsaturated fatty acid (g/day) | 177 | 13.6 | ± | 5.0 |  | 212 | 14.8 | ± | 5.7 | **0.030** |  |
| Cholesterol (mg/day) | 177 | 313 | ± | 135 |  | 212 | 353 | ± | 145 | **0.010** |  |
| Sodium (mg/day) | 177 | 4588 | ± | 1806 |  | 212 | 4635 | ± | 1773 | 0.954 |  |
| Potassium (mg/day) | 177 | 2662 | ± | 931 |  | 212 | 2939 | ± | 1028 | **0.009** |  |
| Calcium (mg/day) | 177 | 670 | ± | 246 |  | 212 | 782 | ± | 289 | **<0.001** |  |
| Magnesium (mg/day) | 177 | 284 | ± | 90 |  | 212 | 309 | ± | 99 | **0.014** |  |
| Iron (mg/day) | 177 | 8.48 | ± | 2.89 |  | 212 | 9.54 | ± | 3.63 | **0.003** |  |
| Vitamin C (mg/day) | 177 | 103 | ± | 49 |  | 212 | 122 | ± | 56 | **0.001** |  |

METs, metabolic equivalents; SD, standard deviation. Values are presented as mean ± SD or n (%). Intergroup differences in the ratios of smokers/non-smokers, alcohol consumers/non-consumers, and prevalence of disease were assessed by χ^2^ tests. Independent differences in the other variables between the groups were assessed using analysis of covariance after adjusting for age. Differences in age between groups were assessed using t-tests.

Supplementary Table S4. Characteristics of men consuming LcS products <3 or ≥3 days/week over the past 10 years

|  | <3 days/week | | | |  | ≥3 days/week | | | |  |
| --- | --- | --- | --- | --- | --- | --- | --- | --- | --- | --- |
|  | n | Mean ± SD | | |  | n | Mean ± SD | | | p value |
| Anthropometric parameters |  |  |  |  |  |  |  |  |  |  |
| Age | 454 | 73.9 | ± | 6.6 |  | 63 | 77.0 | ± | 7.3 | **0.002** |
| Height (m) | 454 | 1.63 | ± | 0.07 |  | 63 | 1.62 | ± | 0.06 | 0.800 |
| Body mass (kg) | 454 | 61.7 | ± | 8.7 |  | 63 | 60.2 | ± | 9.5 | 0.905 |
| Body mass index (kg/m^2^) | 454 | 23.3 | ± | 2.6 |  | 63 | 23.0 | ± | 3.4 | 0.833 |
| Fat-free mass (kg) | 438 | 48.8 | ± | 5.5 |  | 60 | 47.5 | ± | 5.4 | 0.656 |
| Muscle mass (kg) | 438 | 46.2 | ± | 5.2 |  | 60 | 45.0 | ± | 5.1 | 0.658 |
| Current smoking and drinking habits |  |  |  |  |  |  |  |  |  |  |
| Smoker (number [%]) | 454 | 58 |  | [12.8] |  | 63 | 8 |  | [12.7] | 0.986 |
| Alcohol consumer (number [%]) | 454 | 297 |  | [65.4] |  | 63 | 36 |  | [57.1] | 0.199 |
| Physical health measurements |  |  |  |  |  |  |  |  |  |  |
| Systolic blood pressure (mmHg) | 405 | 130.0 | ± | 16.8 |  | 51 | 134.0 | ± | 16.0 | 0.130 |
| Diastolic blood pressure (mmHg) | 405 | 78.6 | ± | 10.5 |  | 51 | 78.5 | ± | 12.0 | 0.404 |
| Preferred walking speed (m/s) | 431 | 1.35 | ± | 0.2 |  | 60 | 1.28 | ± | 0.2 | 0.161 |
| Maximal walking speed (m/s) | 429 | 2.11 | ± | 0.4 |  | 58 | 2.02 | ± | 0.4 | 0.513 |
| Peak handgrip force (N) | 437 | 345 | ± | 73 |  | 60 | 329 | ± | 76 | 0.903 |
| Calcaneal osteosonic index (×10^6^) | 445 | 2.54 | ± | 0.4 |  | 60 | 2.51 | ± | 0.4 | 0.929 |
| Habitual physical activity patterns |  |  |  |  |  |  |  |  |  |  |
| Step count (steps/day) | 244 | 7227 | ± | 3825 |  | 38 | 6696 | ± | 2500 | 0.730 |
| Duration of exercise > 3 METs (min/day) | 244 | 17.7 | ± | 16.7 |  | 38 | 15.8 | ± | 15.0 | 0.794 |
| Blood profile |  |  |  |  |  |  |  |  |  |  |
| Triglyceride (mmol/L) | 383 | 1.54 | ± | 0.90 |  | 47 | 1.64 | ± | 1.03 | 0.345 |
| High-density lipoprotein cholesterol (mmol/L) | 383 | 1.47 | ± | 0.43 |  | 47 | 1.44 | ± | 0.38 | 0.733 |
| Low-density lipoprotein cholesterol (mmol/L) | 383 | 2.87 | ± | 0.76 |  | 47 | 3.04 | ± | 0.70 | 0.060 |
| Glutamic oxaloacetic transaminase (IU/L) | 383 | 25.3 | ± | 17.8 |  | 47 | 24.1 | ± | 7.3 | 0.768 |
| Glutamic pyruvic transaminase (IU/L) | 383 | 21.8 | ± | 18.2 |  | 47 | 19.3 | ± | 7.2 | 0.535 |
| γ-Glutamyl transpeptidase (IU/L) | 383 | 38.5 | ± | 90.5 |  | 47 | 36.1 | ± | 29.5 | 0.917 |
| Glycosylated haemoglobin A1c (%) | 383 | 5.76 | ± | 0.6 |  | 47 | 5.65 | ± | 0.5 | 0.178 |
| Blood sugar (mmol/L) | 383 | 6.63 | ± | 2.04 |  | 47 | 6.57 | ± | 2.07 | 0.467 |
| Albumin (g/L) | 383 | 43.4 | ± | 2.8 |  | 47 | 43.0 | ± | 2.4 | 0.598 |
| Creatinine (μmol/L) | 383 | 82.9 | ± | 29.0 |  | 47 | 82.9 | ± | 20.5 | 0.536 |
| Uric acid (μmol/L) | 383 | 330 | ± | 74 |  | 47 | 344 | ± | 79 | 0.193 |
| Estimated glomerular filtration rate (mL/min/1.73m^2^) | 383 | 65.0 | ± | 14.0 |  | 47 | 64.1 | ± | 15.2 | 0.660 |
| Prevalence |  |  |  |  |  |  |  |  |  |  |
| Hypertension (number [%]) | 454 | 226 |  | [49.8] |  | 63 | 37 |  | [58.7] | 0.183 |
| Dyslipidaemia (number [%]) | 454 | 80 |  | [17.6] |  | 63 | 10 |  | [15.9] | 0.732 |
| Diabetes (number [%]) | 454 | 58 |  | [12.8] |  | 63 | 4 |  | [6.3] | 0.141 |
| Malignant neoplasms (number [%]) | 454 | 47 |  | [10.4] |  | 63 | 9 |  | [14.3] | 0.347 |
| Nutrient intakes |  |  |  |  |  |  |  |  |  |  |
| Energy (kcal/day) | 337 | 2112 | ± | 595 |  | 50 | 2248 | ± | 617 | 0.243 |
| Protein (g/day) | 337 | 74.2 | ± | 23.9 |  | 50 | 83.0 | ± | 33.0 | 0.073 |
| Lipid (g/day) | 337 | 66.4 | ± | 25.0 |  | 50 | 73.5 | ± | 35.0 | 0.162 |
| Carbohydrate (g/day) | 337 | 280 | ± | 79 |  | 50 | 287 | ± | 78 | 0.842 |
| Dietary fiber (g/day) | 337 | 16.2 | ± | 5.5 |  | 50 | 17.1 | ± | 5.3 | 0.523 |
| Saturated fatty acid (g/day) | 337 | 20.4 | ± | 8.2 |  | 50 | 22.5 | ± | 11.4 | 0.206 |
| Monounsaturated fatty acid (g/day) | 337 | 22.2 | ± | 8.8 |  | 50 | 24.6 | ± | 13.5 | 0.156 |
| Polyunsaturated fatty acid (g/day) | 337 | 14.1 | ± | 5.3 |  | 50 | 15.3 | ± | 6.3 | 0.230 |
| Cholesterol (mg/day) | 337 | 330 | ± | 137 |  | 50 | 363 | ± | 167 | 0.427 |
| Sodium (mg/day) | 337 | 4548 | ± | 1776 |  | 50 | 5009 | ± | 1839 | 0.244 |
| Potassium (mg/day) | 337 | 2787 | ± | 988 |  | 50 | 2969 | ± | 1037 | 0.426 |
| Calcium (mg/day) | 337 | 720 | ± | 267 |  | 50 | 793 | ± | 324 | 0.231 |
| Magnesium (mg/day) | 337 | 294 | ± | 94 |  | 50 | 318 | ± | 105 | 0.192 |
| Iron (mg/day) | 337 | 8.92 | ± | 3.26 |  | 50 | 9.90 | ± | 3.83 | 0.171 |
| Vitamin C (mg/day) | 337 | 112 | ± | 53 |  | 50 | 119 | ± | 54 | 0.631 |

LcS, *Lacticaseibacillus* *paracasei* strain Shirota; METs, metabolic equivalents; SD, standard deviation. Values are presented as mean ± SD or n (%). Intergroup differences in the ratios of smokers/non-smokers, alcohol consumers/non-consumers, and prevalence of disease were assessed by χ^2^ tests. Independent differences in the other variables between the groups were assessed using analysis of covariance after adjusting for age. Differences in age between groups were assessed using t-tests.

Supplementary Table S5. Follow-up period in men consuming LcS and overall fermented milk products for <3 or ≥3 days/week over the past 5 years

|  | <3 days/week | |  | ≥3 days/week | | p value |
| --- | --- | --- | --- | --- | --- | --- |
|  | n | Median (month)  [interquartile range] |  | n | Median (month)  [interquartile range] |  |
| LcS products | 401 | 114.08 [90.08, 126.12] |  | 118 | 115.84 [90.09, 138.12] | 0.280 |
| Overall fermented milk products | 246 | 114.08 [90.08, 126.12] |  | 273 | 114.08 [90.08, 138.12] | 0.165 |

Differences between groups were assessed using Mann-Whitney U test.

Supplementary Table S6. Risk of all-cause mortality in men consuming LcS and overall fermented milk products for <3 or ≥3 days/week over the past 10 years

|  | Number of participants | Number of deaths | Mortality | Fisher’s test | Cox model | | |  |
| --- | --- | --- | --- | --- | --- | --- | --- | --- |
|  |  |  | (%) | p value | Hazard Ratio | 95% Confidence interval | p value |  |
| **Full sample ^a^** | | | | | | | | |
| LcS products over the past 10 years | | | | | | | | |
| <3 days/week | 454 | 127 | 28.0 | - | 1.000 | - | - |  |
| ≥3 days/week | 63 | 18 | 28.6 | 1.000 | 0.713 | [0.432–1.177] | 0.186 |  |
| Overall fermented milk products over the past 10 years | | | | | | | | |
| <3 days/week | 355 | 105 | 29.6 | - | 1.000 | - | - |  |
| ≥3 days/week | 162 | 40 | 24.7 | 0.291 | 0.735 | [0.509–1.063] | 0.102 |  |
| **Matched subsample ^b^** | | | | | | | | |
| LcS products over the past 10 years | | | | | | | | |
| <3 days/week | 61 | 21 | 34.4 | - | 1.000 | - | - |  |
| ≥3 days/week | 61 | 16 | 26.2 | 0.431 | 0.742 | [0.457–1.206] | 0.229 |  |
| Overall fermented milk products over the past 10 years | | | | | | | | |
| <3 days/week | 160 | 50 | 32.5 | - | 1.000 | - | - |  |
| ≥3 days/week | 160 | 39 | 24.4 | 0.137 | 0.751 | [0.499–1.133] | 0.172 |  |

LcS, *Lacticaseibacillus* *paracasei* strain Shirota. ^a^ Intergroup differences in the relative risk of all-cause mortality were assessed using Cox proportional hazards regression analysis adjusted for age, body mass index, smoking status, and alcohol consumption. ^b^ Intergroup differences in the relative risk of all-cause mortality were assessed using unadjusted Cox proportional hazards models with robust standard errors clustered by matching sets.

Supplementary Table S7. Risk of all-cause mortality in men according to intake frequency of LcS and overall fermented milk products (continuous variable) over the past 5 years

|  | Number of participants | Cox model ^a^ | | | |
| --- | --- | --- | --- | --- | --- |
|  |  | Hazard Ratio | 95% Confidence interval | p value |  |
| LcS products (days/week) | 519 | 0.931 | [0.872–0.995] | **0.035** |  |
| Overall fermented milk products (days/week) | 519 | 0.926 | [0.877–0.977] | **0.005** |  |

LcS, *Lacticaseibacillus* *paracasei* strain Shirota. ^a^ Intergroup differences in the relative risk of all-cause mortality were assessed using Cox proportional hazards regression analysis adjusted for age, body mass index, smoking status, and alcohol consumption.

Supplementary Table S8. Additional sensitivity analyses of various confounding factors considering the risk of all-cause mortality in men consuming LcS products for <3 or ≥3 days per week over the past 5 years ^1^

|  | Intake frequency | Number of participants | Cox model |  |  |
| --- | --- | --- | --- | --- | --- |
|  |  |  | Hazard ratio | 95% Confidence interval | p value |
| Model 1 | <3 days/week | 401 | 1.000 | – | – |
|  | ≥3 days/week | 118 | 0.648 | [0.438–0.958] | **0.030** |
| Model 2 | <3 days/week | 401 | 1.000 | – | – |
|  | ≥3 days/week | 118 | 0.654 | [0.441–0.970] | **0.035** |
| Model 3 | <3 days/week | 323 | 1.000 | – | – |
|  | ≥3 days/week | 87 | 0.511 | [0.291–0.899] | **0.020** |
| Model 4 | <3 days/week | 250 | 1.000 | – | – |
|  | ≥3 days/week | 68 | 0.644 | [0.358–1.158] | 0.141 |
| Model 5 | <3 days/week | 250 | 1.000 | – | – |
|  | ≥3 days/week | 68 | 0.655 | [0.362–1.182] | 0.160 |
| Model 6 | <3 days/week | 166 | 1.000 | – | – |
|  | ≥3 days/week | 48 | 0.381 | [0.169–0.855] | **0.019** |
| Model 7 | <3 days/week | 136 | 1.000 | – | – |
|  | ≥3 days/week | 39 | 0.503 | [0.217–1.169] | 0.110 |

LcS, *Lacticaseibacillus* *paracasei* strain Shirota. ^1^ Intergroup differences in the relative risk were assessed by Cox proportional hazards regression analyses adjusted for the following confounding factors:

Model 1: age, body mass index, smoking status, alcohol intake

Model 2: age, body mass index, smoking status, alcohol intake, diabetes

Model 3: age, body mass index, smoking status, alcohol intake, muscle mass, preferred walking speed, osteosonic index, glycosylated haemoglobin A1c, diabetes

Model 4: age, body mass index, smoking status, alcohol intake, muscle mass, preferred walking speed, osteosonic index, glycosylated haemoglobin A1c, diabetes, excludes participants with missing dietary intake data

Model 5: age, body mass index, smoking status, alcohol intake, muscle mass, preferred walking speed, osteosonic index, glycosylated haemoglobin A1c, diabetes, energy intake

Model 6: age, body mass index, smoking status, alcohol intake, preferred walking speed, step, estimated glomerular filtration rate, albumin, diabetes, hypertension

Model 7: age, body mass index, smoking status, alcohol intake, preferred walking speed, step, estimated glomerular filtration rate, albumin, diabetes, hypertension, energy intake, protein intake, dietary fiber intake

Supplementary Table S9. Risk of all-cause mortality in men after excluding early deaths, according to intake frequency of LcS and overall fermented milk products (<3 or ≥3 days/week) over the past 5 years

|  | Number of participants | Cox model ^a^ | | | |
| --- | --- | --- | --- | --- | --- |
|  |  | Hazard Ratio | 95% Confidence interval | p value |  |
| Excluding follow-up < 12 months (3 participants were excluded) | |  |  |  |  |
| LcS products over the past 5 years |  |  |  |  |  |
| <3 days/week | 398 | 1.000 | - | - |  |
| ≥3 days/week | 118 | 0.668 | [0.451–0.989] | **0.044** |  |
| Overall fermented milk products over the past 5 years | |  |  |  |  |
| <3 days/week | 272 | 1.000 | - | - |  |
| ≥3 days/week | 244 | 0.697 | [0.502–0.967] | **0.031** |  |
| Excluding follow-up < 24 months (16 participants were excluded) | |  |  |  |  |
| LcS products over the past 5 years |  |  |  |  |  |
| <3 days/week | 388 | 1.000 | - | - |  |
| ≥3 days/week | 115 | 0.667 | [0.443–1.005] | 0.053 |  |
| Overall fermented milk products over the past 5 years | |  |  |  |  |
| <3 days/week | 264 | 1.000 | - | - |  |
| ≥3 days/week | 239 | 0.668 | [0.474–0.941] | **0.021** |  |

LcS, *Lacticaseibacillus* *paracasei* strain Shirota. ^a^ Intergroup differences in the relative risk of all-cause mortality were assessed using Cox proportional hazards regression analysis adjusted for age, body mass index, smoking status, and alcohol consumption.

Supplementary Table S10. Additional sensitivity analyses of various confounding factors considering the risk of all-cause mortality in men consuming overall fermented milk products for <3 or ≥3 days per week over the past 5 years ^1^

|  | Intake frequency | Number of participants | Cox model |  |  |
| --- | --- | --- | --- | --- | --- |
|  |  |  | Hazard ratio | 95% Confidence interval | p value |
| Model 1 | <3 days/week | 246 | 1.000 | – | – |
|  | ≥3 days/week | 273 | 0.690 | [0.499–0.955] | **0.025** |
| Model 2 | <3 days/week | 246 | 1.000 | – | – |
|  | ≥3 days/week | 273 | 0.696 | [0.502–0.966] | **0.030** |
| Model 3 | <3 days/week | 202 | 1.000 | – | – |
|  | ≥3 days/week | 208 | 0.761 | [0.492–1.177] | 0.219 |
| Model 4 | <3 days/week | 148 | 1.000 | – | – |
|  | ≥3 days/week | 170 | 0.871 | [0.541–1.403] | 0.571 |
| Model 5 | <3 days/week | 148 | 1.000 | – | – |
|  | ≥3 days/week | 170 | 0.880 | [0.546–1.418] | 0.599 |
| Model 6 | <3 days/week | 99 | 1.000 | – | – |
|  | ≥3 days/week | 115 | 0.591 | [0.326–1.069] | 0.082 |
| Model 7 | <3 days/week | 77 | 1.000 | – | – |
|  | ≥3 days/week | 98 | 0.696 | [0.368–1.315] | 0.264 |

^1^ Intergroup differences in the relative risk were assessed by Cox proportional hazards regression analyses adjusted for the following confounding factors:

Model 1: age, body mass index, smoking status, alcohol intake

Model 2: age, body mass index, smoking status, alcohol intake, diabetes

Model 3: age, body mass index, smoking status, alcohol intake, muscle mass, preferred walking speed, osteosonic index, glycosylated haemoglobin A1c, diabetes

Model 4: age, body mass index, smoking status, alcohol intake, muscle mass, preferred walking speed, osteosonic index, glycosylated haemoglobin A1c, diabetes, excludes participants with missing dietary intake data

Model 5: age, body mass index, smoking status, alcohol intake, muscle mass, preferred walking speed, osteosonic index, glycosylated haemoglobin A1c, diabetes, energy intake

Model 6: age, body mass index, smoking status, alcohol intake, preferred walking speed, step, estimated glomerular filtration rate, albumin, diabetes, hypertension

Model 7: age, body mass index, smoking status, alcohol intake, preferred walking speed, step, estimated glomerular filtration rate, albumin, diabetes, hypertension, energy intake, protein intake, dietary fiber intake

Supplementary Table S11. Restricted mean survival time in men consuming LcS and overall fermented milk products for <3 or ≥3 days/week over the past 10 years

|  | Participants | Mean difference in survival (months)  (≥3 days/week − <3 days/week) | 95% Confidence interval | p value |
| --- | --- | --- | --- | --- |
| **Full sample ^a^** |  |  |  |  |
| LcS products | 517 | 1.64 | [–7.02 to 10.31] | 0.710 |
| Overall fermented milk products | 517 | 3.97 | [–2.04 to 9.98] | 0.195 |
| **Matched subsample ^b^** |  |  |  |  |
| LcS products | 122 | 3.71 | [–8.11 to 15.54] | 0.538 |
| Overall fermented milk products | 320 | 4.42 | [–2.84 to 11.68] | 0.232 |

LcS, *Lacticaseibacillus* *paracasei* strain Shirota. ^a^ Intergroup differences in the restricted mean survival time up to 138.11 months, corresponding to the maximum follow-up period, were assessed after adjusting for age, body mass index, smoking status, and alcohol consumption. ^b^ Intergroup differences in the restricted mean survival time up to 138.11 months were assessed using an unadjusted RMST model

Supplementary Table S12. Standardised mean differences (unadjusted vs. matched) in men consuming fermented milk products <3 or ≥3 days/week over the past 5 years

|  | Unadjusted | | |  | | Matched | | | |
| --- | --- | --- | --- | --- | --- | --- | --- | --- | --- |
| variable | Mean in <3 group | Mean in ≥3 group | SMD | |  | | Mean in <3 group | Mean in ≥3 group | SMD |
| LcS products | (n = 401) | (n = 118) |  | |  | | (n = 112) | (n = 112) |  |
| Age | 73.4 | 76.5 | 0.444 | |  | | 75.9 | 75.9 | −0.005 |
| BMI | 23.3 | 22.9 | −0.148 | |  | | 23.0 | 22.9 | −0.047 |
| Alcohol | 0.656 | 0.602 | −0.112 | |  | | 0.607 | 0.616 | 0.019 |
| Smoking | 0.132 | 0.110 | −0.067 | |  | | 0.116 | 0.107 | −0.027 |
| Overall fermented milk products | (n = 246) | (n = 273) |  | |  | | (n = 227) | (n = 227) |  |
| Age | 73.7 | 74.5 | 0.118 | |  | | 74.0 | 74.3 | 0.035 |
| BMI | 23.3 | 23.2 | −0.043 | |  | | 23.3 | 23.2 | −0.023 |
| Alcohol | 0.654 | 0.634 | −0.043 | |  | | 0.643 | 0.661 | 0.037 |
| Smoking | 0.167 | 0.092 | −0.225 | |  | | 0.097 | 0.097 | 0.000 |

BMI, body mass index; LcS, *Lacticaseibacillus* *paracasei* strain Shirota; SMD, standardised mean difference.

Supplementary Table S13. Comparison of characteristics between full sample and propensity score–matched subsample among men according to intake frequency of LcS products over the past 5 years

|  | Full sample | | | |  | Matched subsample | | | |  | |
| --- | --- | --- | --- | --- | --- | --- | --- | --- | --- | --- | --- |
|  | n | Mean ± SD | | |  | n | Mean ± SD | | | p value |  |
| Anthropometric parameters |  |  |  |  |  |  |  |  |  |  |  |
| Age | 519 | 74.3 | ± | 6.8 |  | 224 | 76.1 | ± | 6.7 | **<0.001** |  |
| Height (m) | 519 | 1.63 | ± | 0.07 |  | 224 | 1.62 | ± | 0.07 | 0.545 |  |
| Body mass (kg) | 519 | 61.5 | ± | 8.9 |  | 224 | 60.5 | ± | 8.8 | 0.799 |  |
| Body mass index (kg/m^2^) | 519 | 23.2 | ± | 2.7 |  | 224 | 22.9 | ± | 2.6 | 0.457 |  |
| Fat-free mass (kg) | 500 | 48.6 | ± | 5.5 |  | 213 | 47.8 | ± | 5.6 | 0.564 |  |
| Muscle mass (kg) | 500 | 46.1 | ± | 5.2 |  | 213 | 45.3 | ± | 5.3 | 0.564 |  |
| Current smoking and drinking habits |  |  |  |  |  |  |  |  |  |  |  |
| Smoker (number [%]) | 519 | 66 |  | [12.7] |  | 224 | 25 |  | [11.2] | 0.553 |  |
| Alcohol consumer (number [%]) | 519 | 334 |  | [64.4] |  | 224 | 137 |  | [61.2] | 0.407 |  |
| Physical health measurements |  |  |  |  |  |  |  |  |  |  |  |
| Systolic blood pressure (mmHg) | 458 | 130.6 | ± | 16.9 |  | 187 | 130.5 | ± | 17.8 | 0.870 |  |
| Diastolic blood pressure (mmHg) | 458 | 78.6 | ± | 10.7 |  | 187 | 77.7 | ± | 11.5 | 0.881 |  |
| Preferred walking speed (m/s) | 493 | 1.34 | ± | 0.2 |  | 211 | 1.33 | ± | 0.2 | 0.888 |  |
| Maximal walking speed (m/s) | 489 | 2.10 | ± | 0.4 |  | 209 | 2.05 | ± | 0.4 | 0.725 |  |
| Peak handgrip force (N) | 499 | 343 | ± | 74 |  | 214 | 338 | ± | 72 | 0.435 |  |
| Calcaneal osteosonic index (×10^6^) | 507 | 2.54 | ± | 0.4 |  | 216 | 2.51 | ± | 0.4 | 0.903 |  |
| Habitual physical activity patterns |  |  |  |  |  |  |  |  |  |  |  |
| Step count (steps/day) | 284 | 7125 | ± | 3683 |  | 132 | 6594 | ± | 3346 | 0.351 |  |
| Duration of exercise > 3 METs (min/day) | 284 | 17.3 | ± | 16.5 |  | 132 | 14.9 | ± | 14.6 | 0.288 |  |
| Blood profile |  |  |  |  |  |  |  |  |  |  |  |
| Triglyceride (mmol/L) | 432 | 1.55 | ± | 0.91 |  | 174 | 1.51 | ± | 0.91 | 0.922 |  |
| High-density lipoprotein cholesterol (mmol/L) | 432 | 1.47 | ± | 0.42 |  | 174 | 1.44 | ± | 0.36 | 0.506 |  |
| Low-density lipoprotein cholesterol (mmol/L) | 432 | 2.89 | ± | 0.76 |  | 174 | 2.89 | ± | 0.73 | 0.713 |  |
| Glutamic oxaloacetic transaminase (IU/L) | 432 | 25.1 | ± | 17.0 |  | 174 | 23.7 | ± | 6.7 | 0.355 |  |
| Glutamic pyruvic transaminase (IU/L) | 432 | 21.5 | ± | 17.3 |  | 174 | 19.7 | ± | 7.5 | 0.338 |  |
| γ-Glutamyl transpeptidase (IU/L) | 432 | 38.1 | ± | 85.7 |  | 174 | 32.0 | ± | 25.6 | 0.527 |  |
| Glycosylated haemoglobin A1c (%) | 432 | 5.75 | ± | 0.6 |  | 174 | 5.77 | ± | 0.6 | 0.833 |  |
| Blood sugar (mmol/L) | 432 | 6.62 | ± | 2.04 |  | 174 | 6.66 | ± | 2.01 | 0.835 |  |
| Albumin (g/L) | 432 | 43.4 | ± | 2.7 |  | 174 | 43.1 | ± | 2.6 | 0.708 |  |
| Creatinine (μmol/L) | 432 | 82.8 | ± | 28.1 |  | 174 | 83.2 | ± | 30.6 | 0.664 |  |
| Uric acid (μmol/L) | 432 | 331 | ± | 75 |  | 174 | 328 | ± | 80 | 0.756 |  |
| Estimated glomerular filtration rate (mL/min/1.73m^2^) | 432 | 64.9 | ± | 14.1 |  | 174 | 64.7 | ± | 14.7 | 0.488 |  |
| Prevalence |  |  |  |  |  |  |  |  |  |  |  |
| Hypertension (number [%]) | 519 | 263 |  | [50.7] |  | 224 | 123 |  | [54.9] | 0.289 |  |
| Dyslipidaemia (number [%]) | 519 | 90 |  | [17.3] |  | 224 | 34 |  | [15.2] | 0.468 |  |
| Diabetes (number [%]) | 519 | 62 |  | [11.9] |  | 224 | 25 |  | [11.2] | 0.760 |  |
| Malignant neoplasms (number [%]) | 519 | 56 |  | [10.8] |  | 224 | 29 |  | [12.9] | 0.397 |  |
| Nutrient intakes |  |  |  |  |  |  |  |  |  |  |  |
| Energy (kcal/day) | 389 | 2130 | ± | 598 |  | 158 | 2137 | ± | 596 | 0.936 |  |
| Protein (g/day) | 389 | 75.4 | ± | 25.3 |  | 158 | 76.0 | ± | 27.6 | 0.899 |  |
| Lipid (g/day) | 389 | 67.4 | ± | 26.5 |  | 158 | 67.7 | ± | 29.2 | 0.910 |  |
| Carbohydrate (g/day) | 389 | 281 | ± | 78 |  | 158 | 281 | ± | 74 | 0.897 |  |
| Dietary fiber (g/day) | 389 | 16.3 | ± | 5.5 |  | 158 | 16.2 | ± | 5.1 | 0.550 |  |
| Saturated fatty acid (g/day) | 389 | 20.7 | ± | 8.7 |  | 158 | 20.5 | ± | 9.4 | 0.690 |  |
| Monounsaturated fatty acid (g/day) | 389 | 22.5 | ± | 9.5 |  | 158 | 22.5 | ± | 10.9 | 0.894 |  |
| Polyunsaturated fatty acid (g/day) | 389 | 14.3 | ± | 5.4 |  | 158 | 14.3 | ± | 5.6 | 0.957 |  |
| Cholesterol (mg/day) | 389 | 335 | ± | 142 |  | 158 | 331 | ± | 153 | 0.420 |  |
| Sodium (mg/day) | 389 | 4613 | ± | 1786 |  | 158 | 4801 | ± | 1857 | 0.492 |  |
| Potassium (mg/day) | 389 | 2813 | ± | 993 |  | 158 | 2765 | ± | 930 | 0.381 |  |
| Calcium (mg/day) | 389 | 731 | ± | 276 |  | 158 | 730 | ± | 273 | 0.624 |  |
| Magnesium (mg/day) | 389 | 297 | ± | 96 |  | 158 | 296 | ± | 93 | 0.626 |  |
| Iron (mg/day) | 389 | 9.06 | ± | 3.35 |  | 158 | 9.17 | ± | 3.58 | 0.931 |  |
| Vitamin C (mg/day) | 389 | 113 | ± | 54 |  | 158 | 111 | ± | 51 | 0.418 |  |

LcS, *Lacticaseibacillus* *paracasei* strain Shirota; METs, metabolic equivalents; SD, standard deviation. Values are presented as mean ± SD or n (%). Intergroup differences in the ratios of smokers/non-smokers, alcohol consumers/non-consumers, and prevalence of disease were assessed by χ^2^ tests. Independent differences in the other variables between the groups were assessed using analysis of covariance after adjusting for age. Differences in age between groups were assessed using t-tests.

Supplementary Table S14. Characteristics of women consuming LcS products <3 or ≥3 days/week over the past 5 years

|  | <3 days/week | | | |  | ≥3 days/week | | | |  |
| --- | --- | --- | --- | --- | --- | --- | --- | --- | --- | --- |
|  | n | Mean ± SD | | |  | n | Mean ± SD | | | p value |
| Anthropometric parameters |  |  |  |  |  |  |  |  |  |  |
| Age | 537 | 72.9 | ± | 6.4 |  | 224 | 74.2 | ± | 6.5 | **0.008** |
| Height (m) | 537 | 1.50 | ± | 0.06 |  | 224 | 1.49 | ± | 0.06 | 0.734 |
| Body mass (kg) | 537 | 50.7 | ± | 8.1 |  | 224 | 51.1 | ± | 7.2 | 0.234 |
| Body mass index (kg/m^2^) | 537 | 22.5 | ± | 3.1 |  | 224 | 22.9 | ± | 2.9 | 0.109 |
| Fat-free mass (kg) | 530 | 35.4 | ± | 3.3 |  | 217 | 35.1 | ± | 3.3 | 0.920 |
| Muscle mass (kg) | 530 | 33.4 | ± | 3.0 |  | 217 | 33.2 | ± | 3.1 | 0.914 |
| Current smoking and drinking habits |  |  |  |  |  |  |  |  |  |  |
| Smoker (number [%]) | 537 | 16 |  | [3.0] |  | 224 | 3 |  | [1.3] | 0.186 |
| Alcohol consumer (number [%]) | 537 | 114 |  | [21.2] |  | 224 | 41 |  | [18.3] | 0.361 |
| Physical health measurements |  |  |  |  |  |  |  |  |  |  |
| Systolic blood pressure (mmHg) | 484 | 127.4 | ± | 16.6 |  | 194 | 128.6 | ± | 17.1 | 0.586 |
| Diastolic blood pressure (mmHg) | 484 | 74.9 | ± | 9.5 |  | 194 | 75.9 | ± | 8.7 | 0.190 |
| Preferred walking speed (m/s) | 515 | 1.36 | ± | 0.2 |  | 211 | 1.34 | ± | 0.2 | 0.628 |
| Maximal walking speed (m/s) | 512 | 1.97 | ± | 0.4 |  | 209 | 1.91 | ± | 0.4 | 0.381 |
| Peak handgrip force (N) | 523 | 218 | ± | 52 |  | 216 | 217 | ± | 49 | 0.543 |
| Calcaneal osteosonic index (×10^6^) | 532 | 2.19 | ± | 0.2 |  | 220 | 2.16 | ± | 0.2 | 0.343 |
| Habitual physical activity patterns |  |  |  |  |  |  |  |  |  |  |
| Step count (steps/day) | 283 | 7163 | ± | 2950 |  | 111 | 6321 | ± | 2752 | **0.023** |
| Duration of exercise > 3 METs (min/day) | 282 | 16.0 | ± | 13.1 |  | 111 | 12.1 | ± | 12.1 | **0.018** |
| Blood profile |  |  |  |  |  |  |  |  |  |  |
| Triglyceride (mmol/L) | 435 | 1.43 | ± | 0.89 |  | 175 | 1.51 | ± | 0.95 | 0.415 |
| High-density lipoprotein cholesterol (mmol/L) | 435 | 1.70 | ± | 0.42 |  | 175 | 1.68 | ± | 0.41 | 0.574 |
| Low-density lipoprotein cholesterol (mmol/L) | 435 | 3.25 | ± | 0.73 |  | 175 | 3.19 | ± | 0.75 | 0.462 |
| Glutamic oxaloacetic transaminase (IU/L) | 435 | 22.9 | ± | 6.1 |  | 175 | 23.1 | ± | 5.5 | 0.761 |
| Glutamic pyruvic transaminase (IU/L) | 435 | 17.9 | ± | 6.9 |  | 175 | 17.5 | ± | 6.3 | 0.665 |
| γ-Glutamyl transpeptidase (IU/L) | 435 | 23.1 | ± | 32.5 |  | 175 | 22.1 | ± | 23.0 | 0.708 |
| Glycosylated haemoglobin A1c (%) | 435 | 5.64 | ± | 0.5 |  | 175 | 5.69 | ± | 0.4 | 0.226 |
| Blood sugar (mmol/L) | 435 | 6.00 | ± | 1.43 |  | 175 | 6.27 | ± | 1.55 | 0.061 |
| Albumin (g/L) | 435 | 43.9 | ± | 2.6 |  | 175 | 43.7 | ± | 2.4 | 0.534 |
| Creatinine (μmol/L) | 435 | 60.7 | ± | 10.0 |  | 175 | 62.4 | ± | 10.2 | 0.132 |
| Uric acid (μmol/L) | 435 | 270 | ± | 57 |  | 175 | 271 | ± | 52 | 0.926 |
| Estimated glomerular filtration rate (mL/min/1.73m^2^) | 435 | 65.5 | ± | 11.7 |  | 175 | 63.4 | ± | 11.1 | 0.125 |
| Prevalence |  |  |  |  |  |  |  |  |  |  |
| Hypertension (number [%]) | 537 | 205 |  | [38.2] |  | 224 | 102 |  | [45.5] | 0.059 |
| Dyslipidaemia (number [%]) | 537 | 134 |  | [25.0] |  | 224 | 57 |  | [25.4] | 0.886 |
| Diabetes (number [%]) | 537 | 40 |  | [7.4] |  | 224 | 18 |  | [8.0] | 0.781 |
| Malignant neoplasms (number [%]) | 537 | 44 |  | [8.2] |  | 224 | 20 |  | [8.9] | 0.739 |
| Nutrient intakes |  |  |  |  |  |  |  |  |  |  |
| Energy (kcal/day) | 427 | 1961 | ± | 521 |  | 166 | 1998 | ± | 539 | 0.533 |
| Protein (g/day) | 427 | 73.1 | ± | 22.0 |  | 166 | 75.0 | ± | 23.0 | 0.445 |
| Lipid (g/day) | 427 | 67.2 | ± | 24.0 |  | 166 | 68.3 | ± | 24.1 | 0.692 |
| Carbohydrate (g/day) | 427 | 259 | ± | 67 |  | 166 | 265 | ± | 70 | 0.518 |
| Dietary fiber (g/day) | 427 | 16.6 | ± | 5.1 |  | 166 | 17.0 | ± | 5.4 | 0.450 |
| Saturated fatty acid (g/day) | 427 | 20.6 | ± | 8.0 |  | 166 | 21.1 | ± | 8.1 | 0.636 |
| Monounsaturated fatty acid (g/day) | 427 | 22.4 | ± | 8.4 |  | 166 | 22.6 | ± | 8.2 | 0.892 |
| Polyunsaturated fatty acid (g/day) | 427 | 14.2 | ± | 5.1 |  | 166 | 14.1 | ± | 4.6 | 0.720 |
| Cholesterol (mg/day) | 427 | 330 | ± | 120 |  | 166 | 350 | ± | 114 | 0.093 |
| Sodium (mg/day) | 427 | 4373 | ± | 1625 |  | 166 | 4473 | ± | 1631 | 0.630 |
| Potassium (mg/day) | 427 | 2754 | ± | 863 |  | 166 | 2807 | ± | 847 | 0.587 |
| Calcium (mg/day) | 427 | 723 | ± | 248 |  | 166 | 750 | ± | 239 | 0.312 |
| Magnesium (mg/day) | 427 | 283 | ± | 84 |  | 166 | 288 | ± | 84 | 0.648 |
| Iron (mg/day) | 427 | 8.94 | ± | 3.00 |  | 166 | 9.26 | ± | 3.40 | 0.367 |
| Vitamin C (mg/day) | 427 | 122 | ± | 53 |  | 166 | 126 | ± | 51 | 0.407 |

LcS, *Lacticaseibacillus* *paracasei* strain Shirota; METs, metabolic equivalents; SD, standard deviation. Values are presented as mean ± SD or n (%). Intergroup differences in the ratios of smokers/non-smokers, alcohol consumers/non-consumers, and prevalence of disease were assessed by χ^2^ tests. Independent differences in the other variables between the groups were assessed using analysis of covariance after adjusting for age. Differences in age between groups were assessed using t-tests.

Supplementary Table S15. Characteristics of women consuming overall fermented milk products <3 or ≥3 days/week over the past 5 years

|  | <3 days/week | | | |  | ≥3 days/week | | | |  |
| --- | --- | --- | --- | --- | --- | --- | --- | --- | --- | --- |
|  | n | Mean ± SD | | |  | n | Mean ± SD | | | p value |
| Anthropometric parameters |  |  |  |  |  |  |  |  |  |  |
| Age | 226 | 73.5 | ± | 6.9 |  | 535 | 73.1 | ± | 6.2 | 0.464 |
| Height (m) | 226 | 1.50 | ± | 0.06 |  | 535 | 1.50 | ± | 0.06 | 0.761 |
| Body mass (kg) | 226 | 50.7 | ± | 8.7 |  | 535 | 50.9 | ± | 7.5 | 0.877 |
| Body mass index (kg/m^2^) | 226 | 22.6 | ± | 3.2 |  | 535 | 22.6 | ± | 3.0 | 0.820 |
| Fat-free mass (kg) | 223 | 35.1 | ± | 3.4 |  | 524 | 35.4 | ± | 3.3 | 0.394 |
| Muscle mass (kg) | 223 | 33.2 | ± | 3.1 |  | 524 | 33.4 | ± | 3.0 | 0.392 |
| Current smoking and drinking habits |  |  |  |  |  |  |  |  |  |  |
| Smoker (number [%]) | 226 | 8 |  | [3.5] |  | 535 | 11 |  | [2.1] | 0.231 |
| Alcohol consumer (number [%]) | 226 | 45 |  | [19.9] |  | 535 | 110 |  | [20.6] | 0.839 |
| Physical health measurements |  |  |  |  |  |  |  |  |  |  |
| Systolic blood pressure (mmHg) | 202 | 126.7 | ± | 15.3 |  | 476 | 128.1 | ± | 17.3 | 0.276 |
| Diastolic blood pressure (mmHg) | 202 | 74.9 | ± | 9.1 |  | 476 | 75.3 | ± | 9.4 | 0.601 |
| Preferred walking speed (m/s) | 211 | 1.33 | ± | 0.2 |  | 515 | 1.37 | ± | 0.2 | **0.029** |
| Maximal walking speed (m/s) | 210 | 1.93 | ± | 0.4 |  | 511 | 1.96 | ± | 0.4 | 0.439 |
| Peak handgrip force (N) | 215 | 210 | ± | 56 |  | 524 | 221 | ± | 48 | **0.011** |
| Calcaneal osteosonic index (×10^6^) | 222 | 2.18 | ± | 0.2 |  | 530 | 2.17 | ± | 0.2 | 0.392 |
| Habitual physical activity patterns |  |  |  |  |  |  |  |  |  |  |
| Step count (steps/day) | 115 | 6828 | ± | 2786 |  | 279 | 6966 | ± | 2973 | 0.630 |
| Duration of exercise > 3 METs (min/day) | 115 | 14.8 | ± | 11.5 |  | 278 | 14.9 | ± | 13.4 | 0.997 |
| Blood profile |  |  |  |  |  |  |  |  |  |  |
| Triglyceride (mmol/L) | 186 | 1.43 | ± | 0.94 |  | 424 | 1.47 | ± | 0.89 | 0.655 |
| High-density lipoprotein cholesterol (mmol/L) | 186 | 1.72 | ± | 0.43 |  | 424 | 1.69 | ± | 0.40 | 0.394 |
| Low-density lipoprotein cholesterol (mmol/L) | 186 | 3.30 | ± | 0.78 |  | 424 | 3.20 | ± | 0.72 | 0.129 |
| Glutamic oxaloacetic transaminase (IU/L) | 186 | 22.8 | ± | 6.6 |  | 424 | 23.0 | ± | 5.6 | 0.708 |
| Glutamic pyruvic transaminase (IU/L) | 186 | 17.5 | ± | 7.0 |  | 424 | 17.9 | ± | 6.6 | 0.484 |
| γ-Glutamyl transpeptidase (IU/L) | 186 | 23.1 | ± | 36.4 |  | 424 | 22.6 | ± | 26.8 | 0.845 |
| Glycosylated haemoglobin A1c (%) | 186 | 5.69 | ± | 0.6 |  | 424 | 5.64 | ± | 0.4 | 0.254 |
| Blood sugar (mmol/L) | 186 | 6.15 | ± | 1.80 |  | 424 | 6.05 | ± | 1.30 | 0.416 |
| Albumin (g/L) | 186 | 43.8 | ± | 2.7 |  | 424 | 43.9 | ± | 2.5 | 0.959 |
| Creatinine (μmol/L) | 186 | 60.0 | ± | 10.3 |  | 424 | 61.7 | ± | 9.9 | **0.043** |
| Uric acid (μmol/L) | 186 | 267 | ± | 58 |  | 424 | 272 | ± | 55 | 0.309 |
| Estimated glomerular filtration rate (mL/min/1.73m^2^) | 186 | 66.5 | ± | 12.3 |  | 424 | 64.2 | ± | 11.2 | **0.015** |
| Prevalence |  |  |  |  |  |  |  |  |  |  |
| Hypertension (number [%]) | 226 | 99 |  | [43.8] |  | 535 | 208 |  | [38.9] | 0.206 |
| Dyslipidaemia (number [%]) | 226 | 53 |  | [23.5] |  | 535 | 138 |  | [25.8] | 0.496 |
| Diabetes (number [%]) | 226 | 18 |  | [8.0] |  | 535 | 40 |  | [7.5] | 0.817 |
| Malignant neoplasms (number [%]) | 226 | 17 |  | [7.5] |  | 535 | 47 |  | [8.8] | 0.566 |
| Nutrient intakes |  |  |  |  |  |  |  |  |  |  |
| Energy (kcal/day) | 177 | 1891 | ± | 516 |  | 416 | 2006 | ± | 527 | **0.012** |
| Protein (g/day) | 177 | 69.6 | ± | 22.5 |  | 416 | 75.4 | ± | 22.0 | **0.003** |
| Lipid (g/day) | 177 | 64.3 | ± | 23.5 |  | 416 | 68.8 | ± | 24.1 | **0.028** |
| Carbohydrate (g/day) | 177 | 252 | ± | 68 |  | 416 | 265 | ± | 68 | **0.024** |
| Dietary fiber (g/day) | 177 | 15.7 | ± | 4.7 |  | 416 | 17.1 | ± | 5.3 | **0.002** |
| Saturated fatty acid (g/day) | 177 | 19.4 | ± | 7.6 |  | 416 | 21.3 | ± | 8.1 | **0.006** |
| Monounsaturated fatty acid (g/day) | 177 | 21.8 | ± | 8.3 |  | 416 | 22.8 | ± | 8.4 | 0.147 |
| Polyunsaturated fatty acid (g/day) | 177 | 13.9 | ± | 5.3 |  | 416 | 14.3 | ± | 4.9 | 0.308 |
| Cholesterol (mg/day) | 177 | 314 | ± | 121 |  | 416 | 345 | ± | 117 | **0.003** |
| Sodium (mg/day) | 177 | 4315 | ± | 1758 |  | 416 | 4438 | ± | 1567 | 0.350 |
| Potassium (mg/day) | 177 | 2586 | ± | 832 |  | 416 | 2846 | ± | 858 | **<0.001** |
| Calcium (mg/day) | 177 | 657 | ± | 238 |  | 416 | 761 | ± | 243 | **<0.001** |
| Magnesium (mg/day) | 177 | 268 | ± | 83 |  | 416 | 292 | ± | 84 | **<0.001** |
| Iron (mg/day) | 177 | 8.45 | ± | 3.00 |  | 416 | 9.28 | ± | 3.13 | **0.002** |
| Vitamin C (mg/day) | 177 | 111 | ± | 53 |  | 416 | 128 | ± | 51 | **<0.001** |

METs, metabolic equivalents; SD, standard deviation. Values are presented as mean ± SD or n (%). Intergroup differences in the ratios of smokers/non-smokers, alcohol consumers/non-consumers, and prevalence of disease were assessed by χ^2^ tests. Independent differences in the other variables between the groups were assessed using analysis of covariance after adjusting for age. Differences in age between groups were assessed using t-tests.

Supplementary Table S16. Characteristics of women consuming LcS products <3 or ≥3 days/week over the past 10 years

|  | <3 days/week | | | |  | ≥3 days/week | | | |  |
| --- | --- | --- | --- | --- | --- | --- | --- | --- | --- | --- |
|  | n | Mean ± SD | | |  | n | Mean ± SD | | | p value |
| Anthropometric parameters |  |  |  |  |  |  |  |  |  |  |
| Age | 619 | 73.0 | ± | 6.4 |  | 138 | 74.1 | ± | 6.2 | 0.077 |
| Height (m) | 619 | 1.50 | ± | 0.06 |  | 138 | 1.50 | ± | 0.06 | 0.249 |
| Body mass (kg) | 619 | 50.8 | ± | 8.0 |  | 138 | 51.1 | ± | 7.0 | 0.367 |
| Body mass index (kg/m^2^) | 619 | 22.6 | ± | 3.1 |  | 138 | 22.8 | ± | 2.9 | 0.579 |
| Fat-free mass (kg) | 610 | 35.3 | ± | 3.3 |  | 133 | 35.3 | ± | 3.3 | 0.348 |
| Muscle mass (kg) | 610 | 33.3 | ± | 3.0 |  | 133 | 33.4 | ± | 3.0 | 0.341 |
| Current smoking and drinking habits |  |  |  |  |  |  |  |  |  |  |
| Smoker (number [%]) | 619 | 18 |  | [2.9] |  | 138 | 1 |  | [0.7] | 0.138 |
| Alcohol consumer (number [%]) | 619 | 132 |  | [21.3] |  | 138 | 23 |  | [16.7] | 0.220 |
| Physical health measurements |  |  |  |  |  |  |  |  |  |  |
| Systolic blood pressure (mmHg) | 559 | 127.8 | ± | 16.7 |  | 116 | 127.3 | ± | 17.3 | 0.694 |
| Diastolic blood pressure (mmHg) | 559 | 75.3 | ± | 9.4 |  | 116 | 74.7 | ± | 8.6 | 0.563 |
| Preferred walking speed (m/s) | 592 | 1.36 | ± | 0.2 |  | 130 | 1.33 | ± | 0.2 | 0.456 |
| Maximal walking speed (m/s) | 589 | 1.97 | ± | 0.4 |  | 128 | 1.90 | ± | 0.4 | 0.146 |
| Peak handgrip force (N) | 602 | 218 | ± | 52 |  | 133 | 214 | ± | 44 | 0.733 |
| Calcaneal osteosonic index (×10^6^) | 613 | 2.18 | ± | 0.2 |  | 135 | 2.16 | ± | 0.2 | 0.464 |
| Habitual physical activity patterns |  |  |  |  |  |  |  |  |  |  |
| Step count (steps/day) | 318 | 7095 | ± | 2955 |  | 74 | 6225 | ± | 2675 | **0.025** |
| Duration of exercise > 3 METs (min/day) | 317 | 15.9 | ± | 13.3 |  | 74 | 10.8 | ± | 10.3 | **0.003** |
| Blood profile |  |  |  |  |  |  |  |  |  |  |
| Triglyceride (mmol/L) | 505 | 1.44 | ± | 0.87 |  | 104 | 1.54 | ± | 1.10 | 0.294 |
| High-density lipoprotein cholesterol (mmol/L) | 505 | 1.70 | ± | 0.42 |  | 104 | 1.69 | ± | 0.41 | 0.869 |
| Low-density lipoprotein cholesterol (mmol/L) | 505 | 3.24 | ± | 0.74 |  | 104 | 3.21 | ± | 0.74 | 0.807 |
| Glutamic oxaloacetic transaminase (IU/L) | 505 | 22.9 | ± | 5.9 |  | 104 | 23.3 | ± | 5.9 | 0.542 |
| Glutamic pyruvic transaminase (IU/L) | 505 | 17.8 | ± | 6.7 |  | 104 | 17.4 | ± | 6.6 | 0.571 |
| γ-Glutamyl transpeptidase (IU/L) | 505 | 22.7 | ± | 30.3 |  | 104 | 23.3 | ± | 29.0 | 0.862 |
| Glycosylated haemoglobin A1c (%) | 505 | 5.65 | ± | 0.5 |  | 104 | 5.68 | ± | 0.5 | 0.540 |
| Blood sugar (mmol/L) | 505 | 6.03 | ± | 1.42 |  | 104 | 6.32 | ± | 1.68 | 0.073 |
| Albumin (g/L) | 505 | 43.9 | ± | 2.6 |  | 104 | 43.8 | ± | 2.7 | 0.984 |
| Creatinine (μmol/L) | 505 | 60.7 | ± | 9.9 |  | 104 | 63.6 | ± | 10.6 | **0.011** |
| Uric acid (μmol/L) | 505 | 269 | ± | 56 |  | 104 | 273 | ± | 53 | 0.600 |
| Estimated glomerular filtration rate (mL/min/1.73m^2^) | 505 | 65.4 | ± | 11.6 |  | 104 | 62.3 | ± | 10.8 | **0.017** |
| Prevalence |  |  |  |  |  |  |  |  |  |  |
| Hypertension (number [%]) | 619 | 246 |  | [39.7] |  | 138 | 60 |  | [43.5] | 0.419 |
| Dyslipidaemia (number [%]) | 619 | 158 |  | [25.5] |  | 138 | 33 |  | [23.9] | 0.693 |
| Diabetes (number [%]) | 619 | 45 |  | [7.3] |  | 138 | 13 |  | [9.4] | 0.390 |
| Malignant neoplasms (number [%]) | 619 | 49 |  | [7.9] |  | 138 | 15 |  | [10.9] | 0.259 |
| Nutrient intakes |  |  |  |  |  |  |  |  |  |  |
| Energy (kcal/day) | 483 | 1966 | ± | 538 |  | 107 | 1995 | ± | 477 | 0.713 |
| Protein (g/day) | 483 | 73.4 | ± | 22.4 |  | 107 | 74.8 | ± | 21.7 | 0.673 |
| Lipid (g/day) | 483 | 67.5 | ± | 24.5 |  | 107 | 67.6 | ± | 21.9 | 0.929 |
| Carbohydrate (g/day) | 483 | 260 | ± | 69 |  | 107 | 265 | ± | 64 | 0.529 |
| Dietary fiber (g/day) | 483 | 16.7 | ± | 5.2 |  | 107 | 16.6 | ± | 5.0 | 0.808 |
| Saturated fatty acid (g/day) | 483 | 20.8 | ± | 8.2 |  | 107 | 20.7 | ± | 7.0 | 0.827 |
| Monounsaturated fatty acid (g/day) | 483 | 22.6 | ± | 8.6 |  | 107 | 22.1 | ± | 7.3 | 0.537 |
| Polyunsaturated fatty acid (g/day) | 483 | 14.2 | ± | 5.2 |  | 107 | 13.9 | ± | 4.2 | 0.439 |
| Cholesterol (mg/day) | 483 | 333 | ± | 121 |  | 107 | 346 | ± | 107 | 0.410 |
| Sodium (mg/day) | 483 | 4409 | ± | 1670 |  | 107 | 4356 | ± | 1429 | 0.622 |
| Potassium (mg/day) | 483 | 2775 | ± | 873 |  | 107 | 2743 | ± | 800 | 0.642 |
| Calcium (mg/day) | 483 | 726 | ± | 249 |  | 107 | 749 | ± | 234 | 0.485 |
| Magnesium (mg/day) | 483 | 285 | ± | 86 |  | 107 | 284 | ± | 79 | 0.846 |
| Iron (mg/day) | 483 | 8.98 | ± | 3.04 |  | 107 | 9.31 | ± | 3.47 | 0.435 |
| Vitamin C (mg/day) | 483 | 123 | ± | 53 |  | 107 | 122 | ± | 50 | 0.843 |

LcS, *Lacticaseibacillus* *paracasei* strain Shirota; METs, metabolic equivalents; SD, standard deviation. Values are presented as mean ± SD or n (%). Intergroup differences in the ratios of smokers/non-smokers, alcohol consumers/non-consumers, and prevalence of disease were assessed by χ^2^ tests. Independent differences in the other variables between the groups were assessed using analysis of covariance after adjusting for age. Differences in age between groups were assessed using t-tests.

Supplementary Table S17. Follow-up period in women consuming LcS and overall fermented milk products for <3 or ≥3 days/week over the past 5 years

|  | <3 days/week | |  | ≥3 days/week | | p value |
| --- | --- | --- | --- | --- | --- | --- |
|  | n | Median (month)  [interquartile range] |  | n | Median (month)  [interquartile range] |  |
| LcS products | 537 | 126.12 [102.08, 138.12] |  | 224 | 126.12 [102.08, 138.12] | 0.353 |
| Overall fermented milk products | 226 | 126.12 [102.18, 138.12] |  | 535 | 126.12 [102.08, 138.12] | 0.640 |

Differences between groups were assessed using Mann-Whitney U test.

Supplementary Table S18. Risk of all-cause mortality in women according to intake frequency of LcS and overall fermented milk products (continuous variable) over the past 5 years

|  | Number of participants | Cox model ^a^ | | | |
| --- | --- | --- | --- | --- | --- |
|  |  | Hazard Ratio | 95% Confidence interval | p value |  |
| LcS products (days/week) | 761 | 1.039 | [0.976–1.106] | 0.230 |  |
| Overall fermented milk products (days/week) | 761 | 1.027 | [0.960–1.100] | 0.437 |  |

LcS, *Lacticaseibacillus* *paracasei* strain Shirota. ^a^ Intergroup differences in the relative risk of all-cause mortality were assessed using Cox proportional hazards regression analysis adjusted for age, body mass index, smoking status, and alcohol consumption.

Supplementary Table S19. Standardised mean differences (unadjusted vs. matched) in women consuming fermented milk products <3 or ≥3 days/week over the past 5 years

|  | Unadjusted | | |  | | Matched | | | |
| --- | --- | --- | --- | --- | --- | --- | --- | --- | --- |
| variable | Mean in <3 group | Mean in ≥3 group | SMD | |  | | Mean in <3 group | Mean in ≥3 group | SMD |
| LcS products | (n = 537) | (n = 224) |  | |  | | (n = 223) | (n = 223) |  |
| Age | 72.6 | 74.0 | 0.210 | |  | | 74.0 | 73.9 | −0.015 |
| BMI | 22.5 | 22.9 | 0.125 | |  | | 22.9 | 22.9 | −0.023 |
| Alcohol | 0.212 | 0.183 | −0.074 | |  | | 0.211 | 0.184 | −0.068 |
| Smoking | 0.030 | 0.013 | −0.113 | |  | | 0.018 | 0.013 | −0.031 |
| Overall fermented milk products | (n = 226) | (n = 535) |  | |  | | (n = 220) | (n = 220) |  |
| Age | 73.3 | 72.9 | −0.065 | |  | | 73.1 | 73.3 | 0.040 |
| BMI | 22.6 | 22.6 | 0.019 | |  | | 22.6 | 22.7 | 0.043 |
| Alcohol | 0.199 | 0.206 | 0.016 | |  | | 0.195 | 0.200 | 0.011 |
| Smoking | 0.035 | 0.021 | −0.090 | |  | | 0.027 | 0.027 | 0.000 |

BMI, body mass index; LcS, *Lacticaseibacillus* *paracasei* strain Shirota; SMD, standardised mean difference.

Table S20. Characteristics of men and women in the analysis of fermented milk intake and all-cause mortality

|  | Men | | | |  | Women | | | |  |
| --- | --- | --- | --- | --- | --- | --- | --- | --- | --- | --- |
|  | n | Mean ± SD | | |  | n | Mean ± SD | | | p value |
| Anthropometric parameters |  |  |  |  |  |  |  |  |  |  |
| Age | 519 | 74.3 | ± | 6.8 |  | 761 | 73.3 | ± | 6.4 | **0.005** |
| Body mass index (kg/m^2^) | 519 | 23.2 | ± | 2.7 |  | 761 | 22.6 | ± | 3.1 | **<0.001** |
| Current smoking and drinking habits |  |  |  |  |  |  |  |  |  |  |
| Smoker (number [%]) | 519 | 66 |  | [12.7] |  | 761 | 19 |  | [2.5] | **<0.001** |
| Alcohol consumer (number [%]) | 519 | 334 |  | [64.4] |  | 761 | 155 |  | [20.4] | **<0.001** |
| Prevalence |  |  |  |  |  |  |  |  |  |  |
| Hypertension (number [%]) | 519 | 263 |  | [50.7] |  | 761 | 307 |  | [40.3] | **<0.001** |
| Dyslipidaemia (number [%]) | 519 | 90 |  | [17.3] |  | 761 | 191 |  | [25.1] | **<0.001** |
| Diabetes (number [%]) | 519 | 62 |  | [11.9] |  | 761 | 58 |  | [7.6] | **0.009** |
| Malignant neoplasms (number [%]) | 519 | 56 |  | [10.8] |  | 761 | 64 |  | [8.4] | 0.151 |
| Blood profile |  |  |  |  |  |  |  |  |  |  |
| Triglyceride (mmol/L) | 432 | 1.55 | ± | 0.91 |  | 610 | 1.46 | ± | 0.91 | 0.090 |
| High-density lipoprotein cholesterol (mmol/L) | 432 | 1.47 | ± | 0.42 |  | 610 | 1.70 | ± | 0.41 | **<0.001** |
| Low-density lipoprotein cholesterol (mmol/L) | 432 | 2.89 | ± | 0.76 |  | 610 | 3.23 | ± | 0.74 | **<0.001** |
| Glutamic oxaloacetic transaminase (IU/L) | 432 | 25.1 | ± | 17.0 |  | 610 | 22.9 | ± | 5.9 | **0.003** |
| Glutamic pyruvic transaminase (IU/L) | 432 | 21.5 | ± | 17.3 |  | 610 | 17.8 | ± | 6.7 | **<0.001** |
| γ-Glutamyl transpeptidase (IU/L) | 432 | 38.1 | ± | 85.7 |  | 610 | 22.8 | ± | 30.1 | **<0.001** |
| Glycosylated haemoglobin A1c (%) | 432 | 5.75 | ± | 0.6 |  | 610 | 5.66 | ± | 0.5 | **0.009** |
| Blood sugar (mmol/L) | 432 | 6.62 | ± | 2.04 |  | 610 | 6.08 | ± | 1.47 | **<0.001** |
| Albumin (g/L) | 432 | 43.4 | ± | 2.7 |  | 610 | 43.9 | ± | 2.6 | **0.007** |
| Uric acid (μmol/L) | 432 | 331 | ± | 75 |  | 610 | 270 | ± | 56 | **<0.001** |
| Estimated glomerular filtration rate (mL/min/1.73m^2^) | 432 | 64.9 | ± | 14.1 |  | 610 | 64.9 | ± | 11.6 | 0.509 |

SD, standard deviation. Values are presented as mean ± SD or n (%). Participants included those with available intake frequency data for the past 5 years. Creatinine for which the clinical reference range differed between women and men was excluded from the analysis. Differences in age were assessed using t-tests. Differences in the ratios of smokers/non-smokers, alcohol consumers/non-consumers, and prevalence of disease were assessed by χ2 tests. Independent differences in the other variables between sexes were assessed using analysis of covariance after adjustment for age.

Table S21. Life expectancy and mean age at death

|  | Life expectancy at age 65^a^ | Mean age at death in this study |
| --- | --- | --- |
| Men | 19.60 years (−> 84.60 years) | 86.11 years |
| Women | 24.43 years (−> 89.43 years) | 86.08 years |

^a^ Values were calculated from the 2020 Prefectural Life Tables (Gunma Prefecture).

Table S22. Risk of all-cause mortality in women subsample consuming LcS products for <3 or ≥3 days/week over the past 5 years

|  | Number of  participants | Number of  deaths | Mortality  (%) | Cox model ^a^ | | |
| --- | --- | --- | --- | --- | --- | --- |
|  |  |  |  | Hazard Ratio | 95% Confidence  interval | p value |
| **Women subsample (n = 246)^b^** | | |  | | | |
| LcS products over the past 5 years | | |  | | | |
| <3 days/week | 188 | 31 | 16.5 | 1.000 | - | - |
| ≥3 days/week | 60 | 10 | 16.7 | 0.824 | [0.403–1.686] | 0.597 |

LcS, *Lacticaseibacillus* *paracasei* strain Shirota. ^a^ Intergroup differences in the relative risk of all-cause mortality were assessed using Cox proportional hazards regression analysis adjusted for age, body mass index, smoking status, and alcohol consumption. ^b^ Women subsample selected using propensity score to match with men in terms of age, body mass index, smoking habits, alcohol consumption, and prevalence of hypertension, dyslipidaemia, diabetes and malignant neoplasms.

Table S23. Risk of all-cause mortality in women subsample after excluding early deaths, according to intake frequency of LcS products (<3 or ≥3 days/week) over the past 5 years

|  | Number of  participants | Number of  deaths | Mortality  (%) | Cox model ^a^ | | |
| --- | --- | --- | --- | --- | --- | --- |
|  |  |  |  | Hazard Ratio | 95% Confidence  interval | p value |
| **Women subsample (n = 246)^b^** | | |  | | | |
| **Follow up** ≥ 48 months | | |  | | | |
| <3 days/week | 182 | 26 | 14.3 | 1.000 | - | - |
| ≥3 days/week | 57 | 7 | 12.3 | 0.667 | [0.289–1.541] | 0.343 |
| **Follow up** ≥ 72 months | | |  | | | |
| <3 days/week | 174 | 19 | 11.0 | 1.000 | - | - |
| ≥3 days/week | 56 | 6 | 10.7 | 0.705 | [0.280–1.773] | 0.457 |
| **Follow up** ≥ 96 months | | |  | | | |
| <3 days/week | 148 | 12 | 8.1 | 1.000 | - | - |
| ≥3 days/week | 49 | 3 | 6.1 | 0.477 | [0.133–1.716] | 0.257 |

LcS, *Lacticaseibacillus* *paracasei* strain Shirota. ^a^ Intergroup differences in the relative risk of all-cause mortality were assessed using Cox proportional hazards regression analysis adjusted for age, body mass index, smoking status, and alcohol consumption. ^b^ Women subsample selected using propensity score to match with men in terms of age, body mass index, smoking habits, alcohol consumption, and prevalence of hypertension, dyslipidaemia, diabetes and malignant neoplasms.

Supplementary Fig. S1. Flow diagram of participant selection process.

Supplementary Fig. S2. Unadjusted Kaplan–Meier survival curves for all-cause mortality in men (A, B) and women (C, D) consuming LcS or overall fermented milk products for <3 and ≥3 days/week over the past 5 years.

Comparison between LcS intake groups (A, C) and overall fermented milk intake groups (B, D). “+” symbols on the curves indicate censoring, and the shaded areas represent 95% confidence intervals.

Supplementary Fig. S3. Baseline age distribution in men (A, B) and women (C, D) consuming LcS or overall fermented milk products for <3 and ≥3 days/week.

Supplementary Fig. S4. Kaplan–Meier survival curves for all-cause mortality in men (A, B) and women (C, D) consuming overall fermented milk products for <3 and ≥3 days/week over the past 5 years.

(A, C) Cox model-adjusted Kaplan–Meier survival curves were obtained using Cox proportional hazards models, with age, BMI, smoking status, and alcohol consumption as covariates. (B, D) Kaplan–Meier survival curves in a matched subsample were obtained using propensity score–matching analyses balancing age, BMI, alcohol consumption, and smoking status. “+” symbols on the curves indicate censoring, and the shaded areas represent 95% confidence intervals.

Hazard ratios are shown for ≥3 days/week compared with <3 days/week.

The numbers at risk cannot be directly defined for the adjusted survival curves derived from the multivariable Cox models, as these represent model-based predictions for a hypothetical population.
