# Supplementary material for: Association between habitual intake of fermented milk products containing lactic acid bacteria and all-cause mortality: a community-based cohort prospective study
Source: J Nutr Health Aging. 2026 Aug 18;30(10):100956. doi: 10.1016/j.jnha.2026.100956 (PMC13508645; doi:10.1016/j.jnha.2026.100956)
Supplement: Supplementary file 2 [file mmc2.docx]

**Supplementary Information 1**

**Association between habitual intake of fermented milk products containing lactic acid bacteria and all-cause mortality: a community-based cohort prospective study**

Yukitoshi Aoyagi^a*^

Ryuta Amamoto^b^

Sungjin Park^a^

Kazuhito Shimamoto^b^

Takashi Suwa^b^

Hiroshi Makino^b^

Satoshi Matsubara^b^

^a^ Exercise Sciences Research Group, Tokyo Metropolitan Institute of Gerontology, Itabashi, Tokyo, Japan

^b^ Food Research Department, Yakult Central Institute, Kunitachi, Tokyo, Japan

*Address correspondence to

Yukitoshi Aoyagi, Ph.D.

Exercise Sciences Research Group, Tokyo Metropolitan Institute of Gerontology

35-2 Sakaecho, Itabashi-ku, Tokyo 173-0015, Japan

E-mail: aoyagi@tmig.or.jp; Tel: +81-3-3964-3241 ext. 4263; Fax: +81-3-3579-4776

- Purpose

To assess the validity of the recall-based interview assessment of frequency of LcS products intake, we conducted an analysis in a subsample of participants.

- Material and Methods

Data

The subsample consisted of older adults (n = 114) who participated in the Nakanojo Study for five consecutive years and had repeated measurements of LcS product intake. In this subsample, the intake frequency was assessed annually through an interview, in which the participants were asked to recall their intake over approximately one month before each survey.

Using these data, we calculated the average intake frequency over the five-year period. This value was then compared with the retrospectively reported intake frequency over the preceding five years, which was assessed in the fifth year.

Statistical analysis

All analyses were conducted using R Version 4.4.2 [1], and statistical significance was defined at 0.05.

To evaluate the agreement between the prospectively assessed and retrospectively reported intake frequencies of LcS products, we performed two types of analyses.

First, for the intake frequency expressed as a continuous ordinal variable (0–7 days/week), we assessed the agreement using Spearman’s rank correlation coefficient, weighted kappa, and adjacent agreement. The weighted kappa [2] (quadratic weights) was calculated using the irr::kappa2 function. Adjacent agreement was defined as the proportion of participants whose intake frequencies differed by no more than one day per week between the two measures.

Second, for the categorical classification of intake frequency (<3 days/week vs. ≥3 days/week), we evaluated agreement using the overall agreement, Cohen’s kappa coefficient, and McNemar’s test. Cohen’s kappa (unweighted) was calculated using the irr::kappa2 function. McNemar’s test was used to assess the potential systematic differences between the two classifications. The phi coefficient was calculated to quantify the strength of the association between the binary variables.

- Results and Discussion

A high level of agreement was observed between the intake frequency reconstructed from repeated assessments over five years and the retrospectively reported intake frequency over the same 5-year period, assessed at a single time point.

For the ordinal intake frequency (0–7 days/week), Spearman’s correlation coefficients ranged from 0.80 to 0.85 and weighted kappa values ranged from 0.79 to 0.86, indicating strong to almost perfect agreement. The adjacent agreement ranged from 0.74 to 0.81, suggesting that most discrepancies were limited to within one day per week. There were no substantial differences between men and women.

Supplementary Table S24. Agreement between reconstructed and retrospectively recalled LcS intake frequency over a 5-year period (ordinal scale, 0–7 days/week)

| Group | n | Spearman’s r | Weighted kappa^1^ | Adjacent agreement^2^ |
| --- | --- | --- | --- | --- |
| Total | 114 | 0.82 | 0.83 | 0.75 |
| Men | 52 | 0.80 | 0.79 | 0.81 |
| Women | 62 | 0.85 | 0.86 | 0.74 |

1 Weighted kappa: measure of agreement for ordinal variables that accounts for the degree of disagreement, assigning smaller penalties to differences between closer categories.

2 Adjacent agreement was defined as an agreement within ±1 day/week.

For the categorical classification of intake frequency (<3 days/week vs. ≥3 days/week), the overall agreement was approximately 0.84 and Cohen’s kappa ranged from 0.67 to 0.69, indicating substantial agreement according to commonly used classifications [3]. The phi coefficients were approximately 0.68–0.69, indicating a strong association between the two measures. McNemar’s test was not statistically significant in any group, suggesting no systematic difference between the reconstructed and recall-based classifications. There were no substantial differences between men and women.

Supplementary Table S25. Agreement between reconstructed and retrospectively recalled LcS intake frequency over a 5-year period (categorical classification: <3 vs. ≥3 days/week)

| Group | n | Agreement^1^ | Cohen’s kappa^2^ | McNemar’s p-value^3^ | Phi coefficient^4^ |
| --- | --- | --- | --- | --- | --- |
| Total | 114 | 0.84 | 0.68 | 0.480 | 0.69 |
| Men | 52 | 0.85 | 0.69 | 0.724 | 0.69 |
| Women | 62 | 0.84 | 0.67 | 0.752 | 0.68 |

1 Agreement: overall proportion of concordant classifications.

2 Cohen’s kappa: measure of agreement beyond chance.

3 McNemar’s test: assesses systematic differences between paired classifications.

4 Phi coefficient: measure of association for binary variables.

These findings indicate that the recall-based assessment of the intake frequency of LcS products over a 5-year period shows substantial agreement with the intake frequency reconstructed from repeated short-term assessments and is therefore considered to have acceptable validity in this population.

Limitation

This analysis was conducted in a subset of participants who were able to complete five years of follow-up, which may have introduced selection bias toward healthier individuals. In addition, the validity of the 10-year retrospective assessment could not be evaluated due to the limited sample size and the potential for a stronger healthy participant bias.

- Reference

[1] Ihaka R., Gentleman R. R: a language for data analysis and graphics. Journal of Computational and Graphical Statistics 1996;5:299–314. https://doi.org/10.1080/10618600.1996.10474713.
[2] Cohen J. Weighted kappa: nominal scale agreement with provision for scaled disagreement or partial credit. Psychological Bulletin 1968;70:213–220. https://doi.org/10.1037/h0026256
[3] Landis J.R., Koch G.G. The measurement of observer agreement for categorical data. Biometrics 1977;33:159–174. https://doi.org/10.2307/2529310
